# Proteasome mutations associated with CANDLE syndrome cause altered neuronal development by dysregulating polyamine synthesis

**DOI:** 10.1172/JCI199140

**Published:** 2026-08-04

**Authors:** Clayton W. Winkler, Benjamin Schwarz, Katie Williams, Sara Alehashemi, Simote T. Foliaki, Joseph Snow, Lisa Joseph, Audrey Thurm, Christopher L. Friend, Gwendolyn Cooper, Eric Bohrnsen, Farzana Bhuyan, Nathan T. Brandes, Ruin Moaddel, Manfred Boehm, Guibin Chen, Cole D. Kimzey, Bibiana Bielekova, Joanna Kocot, Peter Kosa, Cathryn L. Haigh, Raphaela Goldbach-Mansky, Karin E. Peterson

**Affiliations:** 1 Neuroimmunology Section, Laboratory of Neurological Infections and Immunity (LNII), Rocky Mountain Laboratories (RML), National Institute of Allergy and Infectious Diseases (NIAID),; 2 Research and Technologies Branch, RML, NIAID,; 3 Prion Cell Biology Unit, LNII, RML, NIAID, NIH, Hamilton, Montana, USA.; 4 Translational Autoinflammatory Diseases Section, Laboratory of Clinical Immunology & Microbiology (LCIM), NIAID,; 5 Neuropsychology Consult Service, Intramural Research Program, National Institute of Mental Health (NIMH), and; 6 Neurodevelopmental and Behavioral Phenotyping Service, Intramural Research Program, NIMH, NIH, Bethesda, Maryland, USA.; 7 Laboratory of Clinical Investigation, Biomedical Research Center, National Institute on Aging, NIH, Baltimore, Maryland, USA.; 8 Laboratory of Cardiovascular Regenerative Medicine, Translational Vascular Medicine Branch, National Heart, Lung, and Blood Institute and; 9 Neuroimmunological Diseases Section, LCIM, NIAID, NIH, Bethesda, Maryland, USA.

**Keywords:** Genetics, Metabolism, Neuroscience, Autoimmune diseases, Neurodevelopment

## Abstract

Loss-of-function mutations in PSMB8/β5i and other components of the 20S proteasome result in multiorgan diseases, such as Chronic atypical neutrophilic dermatosis with lipodystrophy and elevated temperature (CANDLE) syndrome. Neurocognitive dysfunction associated with CANDLE suggests that proteasomal mutations may impact neuronal function and development early in life. We generated cerebral organoids (COs) from induced pluripotent stem cells (iPSCs) made from patients with CANDLE. The COs from CANDLE iPSCs exhibited impaired neuronal development when compared with COs from healthy control iPSCs. Impaired neuronal maturation in CANDLE COs was correlated with increased polyamines, which were also elevated in CSF from patients with CANDLE. The proteasome-regulated ornithine decarboxylase (ODC), the rate limiting enzyme in polyamine biosynthesis, was elevated in CANDLE neurons. Inhibition of ODC reversed polyamine overproduction and repaired neuronal maturation in CANDLE COs, suggesting a potential therapeutic avenue for intervention. These findings demonstrate that dysfunction of the proteasome affects neuronal development through overproduction of polyamines via dysregulation of ODC and offer insight into potential therapeutic strategies for CNS-related proteasomal dysfunction.

## Abstract 2

Proteasome dysfunction disrupts neuronal development through excess polyamine production, and inhibiting polyamine production restores neuronal maturation in patient-derived brain organoids.

## Graphical abstract

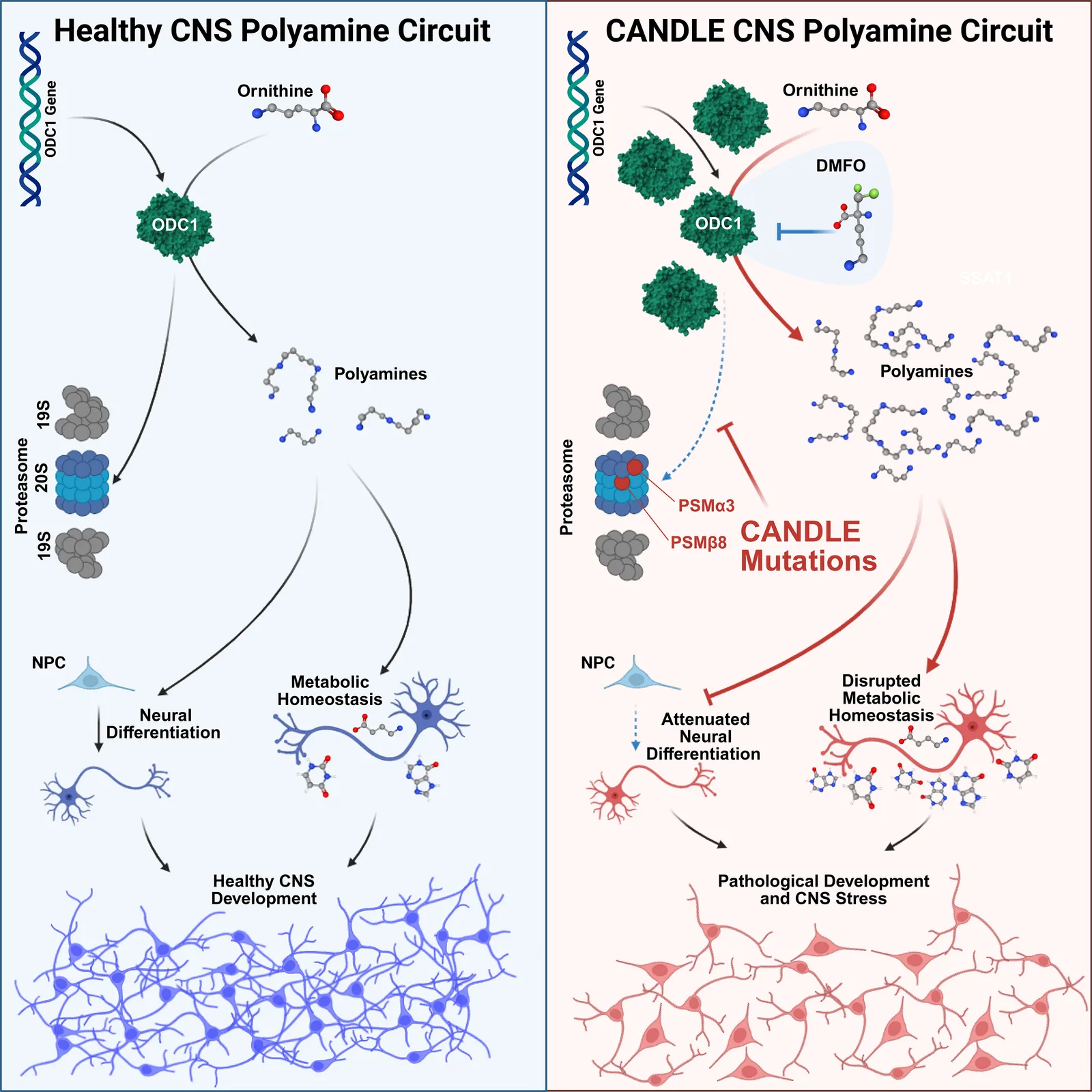

## Introduction

Genetic mutations leading to over activation of innate immune responses can cause autoinflammatory diseases with chronic systemic and organ-specific inflammation. Neurocognitive disorders are increasingly reported in autoinflammatory diseases, suggesting a role for innate immune genes in neurodevelopment. Exploring how these genetic mutations influence the developing central nervous system (CNS) may offer insights into treatments for neurological conditions.

Chronic Atypical Neutrophilic Dermatosis with Lipodystrophy and Elevated temperature (CANDLE), a recently characterized proteasome-associated autoinflammatory syndrome (PRAAS), is most commonly caused by autosomal recessive founder mutations in the proteasome subunit β type-8 gene, *PSMB8*. This gene encodes the inducible β5i subunit, which is incorporated in the 20S immunoproteasome. CANDLE-causing mutations reduce the function of the 20S proteasome core, disrupting intracellular proteolytic degradation (1, 2). Impaired proteostasis results in stress and unfolded protein responses leading to chronic type I IFN production and inflammatory markers in the blood, hallmark features of CANDLE/PRAAS (3, 4). Although rare, CANDLE/PRAAS can also be caused by autosomal recessive loss of function (LOF) mutations in *PSMB9* and *PSMB4* and by digenic recessive or dominant negative forms of inheritance in subunits of the 20S core proteasome or the proteasome assembly proteins *POMP* and *PSMG2* (1, 3, 5, 6).

CANDLE/PRAAS patients typically present in infancy with systemic inflammation, including fevers and rashes due to neutrophilic panniculitis that can lead to lipoatrophy in the extremities as well as face and musculoskeletal symptoms (7). Patients have elevated type I IFN signatures in the blood. Suppression of IFN signaling through treatment with Janus kinase (JAK) inhibitors is effective in controlling inflammatory disease manifestations of CANDLE/PRAAS (7). However, other metabolic features can persist despite treatment (7), suggesting that other mechanisms also contribute to CANDLE/PRAAS. Developmental and cognitive delays were reported in the earliest descriptions of CANDLE/PRAAS, then referred to as Nakajo Nishimura syndrome (8), but severity varied substantially (9). CNS manifestation commonly include basal ganglia calcifications (5, 9–11); and, in rare cases, seizures have been reported (10). Cognitive impairment is a variable manifestation, particularly in children with severe early-onset disease, affecting approximately 42.5% of patients in previously reported cohorts and 66.7% of patients in our cohort (9–11).

Emerging evidence supports a role for the proteasome in neuronal development, including the regulation of axonal regeneration, calcium signaling, neuronal plasticity, and synapse number and function (12–14). Proteasome dysfunction has also been implicated in neurodegenerative diseases. Notably, the *PSMB8-*encoded β5i subunit of the immunoproteasome has been detected in both neurons and neural stem cells (12). More recently, immunoproteasome activity has been identified in neuronal extracellular vesicles of patients with Parkinson’s disease (PD), and *PSMB8*/β5i immunoproteasome activity was observed in dopaminergic neurons in a murine PD model (15). While *PSMB8*/β5i upregulation in neurons following innate immune stimulation, including by type I IFNs, is consistent with the immunoproteasome’s established role in stress and high demand conditions (16), its constitutive expression in developing or differentiating neurons under noninflammatory conditions remains poorly understood.

To study the impact of CANDLE-causing loss of function *PSMB8* mutations on neuronal differentiation and neurodevelopment, we generated cerebral organoids (COs) using iPSCs derived from patients with CANDLE/PRAAS and healthy controls. We utilized early stage (26 day old) COs, as they contain differentiating neuronal precursor cells and developing neurons without confounding cell types, such as glia and endothelial cells, and offer a 3-D in vitro environment to model genetic and molecular mechanisms in neuronal precursors and neuronal differentiation that, when impaired, may cause neurodevelopmental disorders (17, 18). Cellular composition and organization of COs mimic fetal human cortical development (19) and the 3-D structure offers distinct advantages in mapping metabolic interactions under transport-limited conditions and tracing cell migration during neuronal development beyond existing cell culture models (20). Histological and flow cytometric analyses of COs from patients with CANDLE showed fewer neural progenitor cells and mature neurons than healthy controls (HCs), suggesting altered neurodevelopment. These COs, along with cerebral spinal fluid (CSF) from patients with CANDLE and HCs, were then analyzed for metabolic changes to identify mechanisms underlying this altered neurodevelopment.

## Results

### Patients with CANDLE syndrome have low Wechsler Intelligence scores.

Patients diagnosed with CANDLE syndrome have reported CNS-associated pathologies including basal ganglia calcifications, intellectual disability, and, in rare cases, seizures (5, 8–11). We used standardized psychometric testing (21) to assess neurocognition in 6 adolescent or young adult patients with clinically and genetically confirmed CANDLE syndrome. We utilized age- and ability-appropriate Wechsler Intelligence Scale instruments to estimate intellectual abilities (22, 23) in this cohort. CANDLE patients CAN1, CAN2, CAN5 and CAN7 were tested once, while patients CAN3 and CAN4 were tested twice. All 6 patients scored below low average (85 IQ) at all test dates, and 4 of the 6 patients consistently scored below extremely low (≤ 69 IQ) (Figure 1A). These data, along with previous reports, suggest that lower than normal IQ may be a component of CANDLE/PRAAS syndrome.

### COs generated from CANDLE patient iPSCs have altered neurodevelopment.

To assess whether CANDLE/PRAAS-associated *PSMB8* mutations impair neurodevelopment, we generated COs from induced pluripotent stem cells (iPSCs) derived from 3 patients with CANDLE (CAN1, CAN2, CAN3) and 2 healthy controls (HC1 and HC2) (Figure 1B and Supplemental Table 1; supplemental material available online with this article; https://doi.org/10.1172/JCI199140DS1). Organoids were evaluated through day 26 of development, corresponding to approximately 5–8 weeks after conception, a critical developmental window when the cortex has begun to form and any effects of reduced proteasome function on early neuronal differentiation would be detectable. At this stage, COs reproducibly contain a spectrum of neuronal cell types, from progenitors to maturing neurons, prior to the onset of astrocyte differentiation, minimizing confounding effects and allowing for a more accurate assessment of early neuronal differentiation trajectories. All CANDLE patients had *PSMB8* mutations; CAN2 and CAN3 were homozygous or compound heterozygous for pathogenic *PSMB8* variants, and CAN1 had digenic variants in *PSMB8* and *PSMA3* (Supplemental Table 1). Although the iPSC donors for the HC lines were older than the CANDLE donors at the time of iPSC generation, previous studies have shown that the donor age does not significantly affect iPSC differentiation capacity, senescence, or overall functionality (24).

To evaluate the impact of *PSMB8* mutations on early neurodevelopment, we analyzed between 12–22 COs per iPSC line by IHC at 26 days. COs derived from HC donors exhibited typical cellular and structural organization with SOX2^+^ neural progenitors forming rosette-like structures and TUJ1^+^ neuronal projections extending between the progenitor-rich regions, consistent with normal early cortical maturation (Figure 2, A and B). In contrast, COs from all 3 patients with CANDLE showed reduced dispersal of SOX2^+^ rosettes (Figure 2C) and a marked decrease in TUJ1^+^ labeling throughout the organoid (Figure 2D). Quantitative analysis revealed a lower percentage of CO area labeling for SOX2^+^ (Figure 2E) and TUJ1^+^ (Figure 2F) cells in all CANDLE COs compared with HC COs, with each CANDLE CO scoring below the average of either HC. RNA analysis indicated that neuronal differentiation was largely preserved, as *NEUN* mRNA expression was comparable between CANDLE and HC COs. However, reduced *MAP2* mRNA levels in CANDLE COs suggest impaired neuronal maturation and dendritic development (Supplemental Figure 1A). Flow cytometric analysis of dissociated COs from CAN1 and HC1 further confirmed this trend with the proportion of SOX2^+^ and TUJ1^+^ cells being significantly reduced in CAN1-derived COs compared with HC1 (Figure 2G and Supplemental Figure 1, B–G). Collectively, these findings indicated that *PSMB8* mutations disrupt neuronal maturation and organization during early cortical development, suggesting that proteasome dysfunction in CANDLE syndrome may adversely affect neurodevelopmental trajectories.

### CANDLE-causing mutations do not induce dysregulated IFN responses in COs.

In the blood, one of the most notable signatures of CANDLE syndrome is chronic IFN overproduction (7, 25, 26). To investigate the role of IFN on neuronal differentiation in early neuronal development, we analyzed IFN production in HC and CANDLE COs. Surprisingly, no differences in gene expression of *IFNB1* or IFN-stimulated genes (ISGs) *ISG15,* *IFIT1,* and *IFIT2* were observed between CANDLE and HC COs (Figure 3A). Similarly, IFNs and related cytokines were not increased in supernatants from cultured CAN1 COs compared with HC1 COs (Figure 3, B and E), indicating that CANDLE COs did not have elevated IFN responses. We next assessed if activation of CANDLE COs induced high IFN response by inoculating COs with La Crosse virus (LACV) or Inkoo virus (INKV), which are known to infect neurons and induce IFN responses (27). Unexpectedly, CAN1 COs expressed lower *IFNB1* mRNA following virus infection than HC1 COs (Figure 3C). Likewise, the ISG response as determined by *IFIT1* mRNA expression, was similar or lower between HC1 and CAN1 COs under mock or virus infection conditions (Figure 3D). IFN-related proteins IFN-β, IFN-γ, IL-28A, and IL-29 were also not elevated in supernatants from CAN1 compared with HC1 COs either prior to or after virus stimulation (Figure 3, B, E, and F). Thus, the differences in neuronal development observed in CANDLE COs were not associated with elevated IFN responses, suggesting that impaired neuronal maturation is independent of IFN-driven inflammation. Although type I IFN overproduction is a hallmark of the peripheral immune responses in patients with CANDLE (7), this signature was not evident in the CNS-like environment of the COs.

None of the other analytes in the human inflammation panel with detectable protein expression were significantly elevated in CAN1 compared with HC1 COs, indicating that CANDLE-associated mutations did not induce a robust inflammatory response in neuronal cells (Figure 3, E and F). Instead, APRIL (A Proliferation-Inducing Ligand, TNFSF13) and PENT-3 (pentraxin-3), were consistently lower in CAN1 than in HC1 in both unstimulated and virus-infected COs (Figure 3, E and F). APRIL, a tumor necrosis family member associated with immune cell activation, is involved in axon elongation (28). PENT-3, which acts as a chemoattractant and is involved in complement activation, contributes to neurogenesis (29). The lower levels of these proteins in CAN1 compared with HC1 COs (Figure 3, E and F) correlated with the above findings of decreased neuronal maturation in CAN1 COs (Figure 2). Collectively, these findings support the presence of neurodevelopmental abnormalities in CANDLE COs that are independent of overt cytokine dysregulation.

### Metabolic dysregulation in COs from patients with CANDLE.

The impact of proteasome mutations in metabolic pathways in patients with CANDLE, particularly on lipid metabolism in hepatocytes, is well established and clinically evident as dyslipidemia and features of metabolic syndrome (30). These findings suggest that disrupted metabolic homeostasis may also contribute to altered neurodevelopment in CANDLE (5, 7).

To explore this possibility, we profiled metabolites from both COs and their culture supernatants. We compared pooled COs generated from CAN1 and CAN2 with those from HC3. Among the top differentially abundant metabolites in both the cellular (Supplemental Figure 2A) and supernatant (Supplemental Figure 2B) fractions were ornithine, an amino acid and polyamine precursor, and γ-aminobutyric acid (GABA), a neurotransmitter that can be produced downstream of polyamine catabolism (31). Both were elevated in CAN1 and CAN2 COs relative to HC3 COs. The polyamine putrescine was also elevated in the cellular fraction, and spermine levels were increased in the supernatant of CANDLE COs. These changes indicate that polyamine levels are increased in CANDLE COs relative to HC COs, with elevations observed across upstream, intermediate, and downstream metabolites in this pathway.

To expand our analysis of central metabolic pathways in addition to polyamine metabolism, we profiled COs from 5 iPSC lines, HC1, HC2, CAN1, CAN2, and CAN3, using a broader coverage LC-MS/MS method. Univariate analysis across all samples revealed significant metabolic perturbation in CANDLE COs affecting 3 major pathways, including amine metabolism, Acetyl-CoA–associated central metabolic processes, and nucleobase metabolism (Figure 4, A–E). Pathway mapping demonstrated a shift toward increased polyamine production in CANDLE COs (Figure 4B), an expanded pool of nucleobases (Supplemental Figure 3A), and disruption of Acetyl-CoA–generating metabolic pathways (Supplemental Figure 3C).

### Metabolic dysregulation in the CSF of patients with CANDLE.

To determine whether the metabolic alterations observed in CANDLE COs were also present in patients, we analyzed CSF metabolites from 3 patients with CANDLE. One patient (CAN3) overlapped with the CO studies, while the other 2, including CAN6 (with digenic *PSMB4, PSMB9* variants) and CAN7 (harboring dominant-negative *PSMB8* variants) (Figure 1B), did not provide samples for iPSC generation. CANDLE CSF samples were compared with CSF from 7 healthy controls (unrelated to the CO study) and 10 patients with Multiple Sclerosis (MS), serving as an inflammatory CNS disease comparator group (Supplemental Table 2).

Consistent with findings in CANDLE COs, patient CSF samples showed disruptions in the same 3 metabolic pathways — polyamine metabolism (including increased GABAnergic signaling), central energy generating processes related to Acetyl-CoA, and nucleobase metabolism. In fact, these alterations were more pronounced in CSF than in COs, with higher levels of polyamines, GABA, and nucleobases observed in patients with CANDLE (Figure 5, A–E, and Supplemental Figure 3). In contrast, CSF from patients with MS did not show similar metabolic dysregulation (Figure 5, C–E), suggesting that these changes are specific to CANDLE/PRAAS-associated metabolic dysfunction rather than secondary to neuroinflammation. Although polyamine levels vary during development, and 2 of the 3 CANDLE CSF donors were younger than both healthy and MS controls (Supplemental Table 2), the most pronounced elevations in amine and nucleobase metabolites were observed in CAN7, the oldest of the 3 patients. Prior longitudinal studies demonstrate a steep decline in polyamine levels during infancy, followed by relatively stable levels or modest increases from early childhood through adulthood (32, 33). Therefore, age is unlikely to account for the elevated polyamine levels observed in CANDLE CSF relative to HC or MS samples.

### CANDLE mutations impact polyamine metabolism differently in neural progenitors and neuronal cells.

Given the neuronal maturation defects observed in CANDLE COs, we assayed CANDLE and HC iPSCs-derived immature neural progenitor cells (NPCs) and differentiated neurons for the same metabolic alterations. CANDLE NPCs showed significant changes in polyamines, nucleobases, and Acetyl-CoA–related pathways (Figure 6, A and B, Supplemental Figure 3, and Table 1). Thus, CANDLE mutations consistently disrupt the same core pathways across multiple CNS-relevant models. However, unlike COs and CSF, polyamines and nucleobases were downregulated in CANDLE NPCs, (Figure 6, A and B, and Supplemental Figure 3). For example, both putrescine and spermine were decreased, while Acetyl-CoA and N-acetylspermidine were elevated, suggesting increased polyamine acetylation in CANDLE NPCs.

In contrast, differentiated CANDLE neurons exhibited metabolic profiles more similar to COs and CSF, including increased amine metabolites such as GABA and glutamate, elevated nucleobases such as hypoxanthine and uridine, and reduced glycolysis and FAO intermediates (Figure 6, C and D, and Supplemental Figure 3). Thus, CANDLE mutations affected polyamine production differently in NPCs and neurons.

### ODC and PSMB8 expression differs in NPCs, neurons, and COs.

Polyamine synthesis is regulated by the rate limiting enzyme ODC, whose expression is controlled by 20S proteasome-mediated degradation in a negative feed back loop (34). We next examined ODC protein expression in NPCs, differentiated neurons, and COs derived from HC or CANDLE iPSC. ODC expression did not significantly differ between HC NPCs and CANDLE NPCs (Figure 6E and Supplemental Figure 4). In contrast ODC expression was significantly higher in CAN-derived neurons compared with HC-derived neurons (Figure 6E and Supplemental Figure 4). No significant difference was observed in COs, consistently with the lower number of neurons in CANDLE relative to HC COs. These findings suggest that increased ODC expression associated with CANDLE mutations is dependent on neuronal maturation state and correlated with elevated polyamine levels.

To assess whether ODC expression might be influenced by immunoproteasome expression, we examined protein levels of PSMB8/β5i in NPCs, neurons, and COs from CANDLE and HC lines. PSMB8/β5i was below the limit of detection in HC NPCs and only expressed at low levels in 1 of 2 CANDLE lines (Figure 6F and Supplemental Figure 4). In contrast, PSMB8/β5i was expressed in CANDLE and HC neurons as well as HC and CANDLE COs, with inconsistent variation between ratios of immature (I) to mature (M) forms (Figure 6F and Supplemental Figure 4). The detection of PSMB8/β5i in neurons, but not NPCs, correlates with the altered ODC expression in CANDLE neurons but not NPCs. This supports a cell type–specific effect of CANDLE-causing mutations and suggests that altered ODC regulation in CANDLE neurons may be linked to developmental stage–dependent differences in proteasome composition.

### ODC Inhibition in CANDLE COs reduces polyamine levels and promotes neuronal maturation.

To determine whether inhibiting ODC activity could reverse the metabolic and neurodevelopmental abnormalities observed in CANDLE, we treated CANDLE and HC COs with difluoromethylornithine (DFMO), a selective small-molecule inhibitor of ODC. DFMO-containing media or vehicle control was added to COs starting at day 6 and replenished daily for another 20 days. Dose-response analysis showed that DFMO was well tolerated and did not impair organoid growth (Supplemental Figure 5, A and B) with 100μM selected as the highest nontoxic concentration for further studies (Supplemental Figure 5).

Following 20 days of treatment, DFMO-treated CANDLE COs showed a partial reversal of the disease-associated metabolic signatures in amine, Acetyl-CoA, and nucleobase pathways (Figure 7A, Supplemental Figure 6 compared with Supplemental Figure 2). As expected, putrescine levels were significantly reduced in DFMO-treated CANDLE COs relative to vehicle controls (Figure 7B). Metabolites involved in both Acetyl-CoA–related pathways and nucleobase salvage pathways were also markedly decreased, suggesting that altered polyamine metabolism was a key driver of the broader metabolic dysregulation observed in CANDLE models and CSF samples (Figure 7C, Supplemental Figure 6B). Importantly, DFMO-induced metabolic corrections were largely specific to CANDLE COs, with minimal or opposite effects in HC COs, particularly for metabolites such as Acetyl-CoA, FAO intermediates, hypoxanthine, and putrescine (Figure 7, B and C). This differential response suggests that HC COs may possess compensatory mechanisms that buffer against DFMO-induced changes, mechanisms that are unavailable in CANDLE COs.

Beyond direct and proximal metabolic effects, DFMO treatment also rescued the neurodevelopmental phenotype in CANDLE COs. Real-time PCR analysis at day 26 revealed no change in *SOX2* mRNA levels, indicating that progenitor cell populations were unaffected. However, *TUBB3* (TUJ1) mRNA levels were significantly elevated across all DFMO-treated CANDLE lines relative to untreated controls (Supplemental Figure 6D), indicating enhanced neuronal maturation. Analysis of SOX2 and TUJ1 protein expression in vehicle control and DFMO-treated immunolabeled tissues confirmed these results, with SOX2 levels remaining unchanged and TUJ1 expression being increased relative to control (Figure 7, D–G). Thus, blocking polyamine synthesis with DFMO restores neurodevelopmental trajectories specifically in differentiated neurons in the CANDLE CO model. The specificity of DFMO treatment for altering neurodevelopment in differentiated CANDLE neurons correlates with the increased expression of PSMB8/β5i and ODC in the same cells (Figure 6) suggesting that elevated polyamines may be predominately impacting this stage of neurodevelopment.

## Discussion

Our study uncovers a previously unrecognized, cell-type–specific and IFN-independent role for the 20S immunoproteasome in regulating neuronal maturation, driven by CANDLE-causing loss-of-function mutations in *PSMB8*. We demonstrate that impaired immunoproteasome activity leads to the accumulation of ODC in neurons but not NPCs. ODC is a key proteasome-regulated, rate-limiting enzyme in polyamine biosynthesis. This metabolic axis offers a therapeutic target that may help mitigate CNS manifestations in patients with CANDLE/PRAAS. Whether similar proteasome-ODC–polyamine circuits contribute to dysregulated adipocyte or hepatocyte metabolism in these patients remains an open question and warrants further investigation.

Monogenic defects that impair the 20S proteasome produce shared IFN-mediated systemic phenotypes, yet also give rise to disease-specific, tissue-restricted manifestations that point to uncompensated stress pathways operating in distinct cellular contexts. In this study, we demonstrate that CANDLE‑causing *PSMB8* mutations exert a previously underappreciated impact on CNS development, independent of IFN signaling. We establish that PSMB8/β5i is expressed in differentiated neurons and COs and that *PSMB8* CANDLE mutations affect neuronal maturation in COs. It is important to note that COs have limitations in modeling brain development and function, as they do not have the same complexity of structure or cell types. Thus, these findings do not demonstrate that PSMB8/B5i mutations lead to altered brain development or function. However, these studies do identify a direct role for the immunoproteasome in human neurodevelopment, extending its function beyond immune and inflammatory contexts.

Using patient‑derived iPSCs, we show that proteasome impairment leads to accumulation of the rate‑limiting polyamine biosynthetic enzyme ODC, resulting in dysregulated polyamine metabolism (Supplemental Table 3) and impaired neuronal maturation. This mechanism links CANDLE to other monogenic neurodevelopmental disorders characterized by altered polyamine homeostasis (35, 36). Notably, Bachmann-Bupp syndrome, caused by gain‑of‑function mutations in ODC, results in constitutive polyamine overproduction and neurodevelopmental delay (35), and early treatment with DFMO has been shown to improve clinical outcomes (36). Similarly, loss‑of‑function mutations in spermine synthase (SMS) cause Snyder-Robinson syndrome, an X‑linked intellectual disability associated with excessive polyamine synthesis. Polyamines are known to influence development through multiple mechanisms, including nucleic acid binding, chromatin remodeling, RNA regulation, and cell‑cycle control, which meshes with their apparent impact on the CNS (37–39). Together with our findings, these disorders support a requirement for tight regulation of polyamine levels during neurodevelopment (40).

Importantly, DFMO treatment restored neuronal differentiation in CANDLE COs, normalizing not only polyamine levels but also downstream nucleobase and Acetyl‑CoA–related metabolic pathways (Table 1). These findings support polyamine overproduction as an upstream driver of broader metabolic dysregulation in CANDLE. The absence of comparable effects in HC organoids suggests that DFMO selectively corrects disease‑associated metabolic imbalance without disrupting normal homeostasis, highlighting its potential as a tissue‑specific targeted therapeutic strategy for CNS involvement in CANDLE/PRAAS (36). However, the effects of DFMO on neurodevelopment were largely confined to differentiated neurons, despite evidence that NPCs were also impacted by CANDLE mutations in COs. Interestingly, PSMB8/β5i was detectable in differentiated neurons but not in NPCs, suggesting that impaired degradation of ODC by mutant proteasome subunits may preferentially affect cells further along the neuronal differentiation pathway, leading to polyamine accumulation in mature neuronal populations. Given the lack of detectable PSMB8/β5i expression in NPCs, the magnitude of the metabolic phenotype in CANDLE NPCs is unexpected. It is unclear whether this reflects residual PSMB8/β5i expression below detection thresholds or alternative mutation-associated mechanisms independent of immunoproteasome expression. Our data suggest that the NPC metabolic phenotype is likely not primarily polyamine-driven and warrants further mechanistic investigation.

Our findings further distinguish CANDLE‑associated 20S proteasome dysfunction from disorders caused by mutations in the 19S regulatory particle (RP) of the 26S proteasome (26). While 19S RP mutations are associated with severe neurodevelopmental and autism‑spectrum phenotypes in the absence of systemic inflammation, both 19S and 20S defects converge on proteostasis failure in neurons (41–43). ODC provides a mechanistic point of convergence: it can be degraded directly by the 20S proteasome in a ubiquitin‑independent manner (44), while its regulator antizyme depends on 19S‑mediated ubiquitination for turnover (45). Thus, both classes of proteasome defects may dysregulate polyamine synthesis via distinct but intersecting mechanisms, underscoring the need for direct comparative studies.

Across models, including CSF, COs, NPCs, and neurons, we identified 3 recurrent metabolic signatures in CANDLE: amine (polyamine and GABA), nucleobase, and Acetyl‑CoA metabolism (Supplemental Table 3). Although these pathways were consistently affected, the specific metabolites and directionality varied by cell type and compartment (Table 1 and Supplemental Figure 3), likely reflecting differences between intracellular and extracellular environments, blood-brain barrier constraints, and cell‑specific metabolic capacity (46–49). Neurons are highly metabolically restricted and avoid fatty acid oxidation (FAO) and aerobic glycolysis due to vulnerability to oxidative damage (48, 50–52). We hypothesize that this metabolic inflexibility limits their buffering capacity, specifically their ability to generate sufficient acetyl-CoA to support acetylation and inactivation of excess polyamines (Table 1 and Supplemental Figure 3). Although the arginine/ornithine/putrescine (AOP) pathway for de novo GABA synthesis has traditionally been considered astrocyte-restricted, it has also been demonstrated in midbrain dopaminergic neurons, suggesting that differentiated neurons retain conditional access to this pathway (53, 54). Given the observed trend toward increased GABA levels in both CO and neuron cultures, both systems that lack astrocytes, we suspect that ODC dysregulation in neurons may enhance flux through the AOP pathway. However, the functional relevance of this observation in CNS physiology requires further investigation. In contrast to the metabolic rigidity of neurons, NPCs demonstrated greater metabolic flexibility, particularly in acetyl‑CoA–dependent polyamine acetylation, suggesting that cell‑type–specific compensatory capacity may modulate the downstream consequences of ODC dysregulation (Table 1 and Supplemental Figure 3).

Consistent with the brain’s reliance on nucleobase salvage rather than de novo synthesis (55, 56), CANDLE models showed increased hypoxanthine, uridine, cytidine, and uracil levels, likely reflecting heightened nucleotide demand under metabolic stress and increased neuronal activity. Although hypoxanthine has been linked to ROS generation via xanthine oxidoreductase (XOR) in peripheral tissues (57, 58). XOR is minimally expressed in the brain, making ROS-mediated toxicity less likely in this context. Instead, elevated hypoxanthine and related nucleobases likely reflect increased salvage pathway activity in response to metabolic stress and demand for nucleotide triphosphates, particularly ATP, consistent with elevated adenosine levels observed in CANDLE CSF and neurons (58). While a direct mechanistic link between polyamine accumulation and nucleobase metabolism remains to be fully defined, the reduction in nucleobase levels following DFMO treatment supports the model in which excess polyamines drive secondary metabolic stress responses, including enhanced nucleotide recycling.

In conclusion, our multimodel analysis across CSF, cerebral organoids, NPCs, and differentiated neurons demonstrates that impaired 20S proteasome function in CANDLE disrupts neurodevelopment through interconnected metabolic disturbances, including ODC accumulation, elevated polyamine levels, altered nucleobase metabolism, and dysregulated acetyl-CoA pathways. Importantly, these CNS abnormalities can arise independently of canonical IFN signaling, revealing a previously unrecognized, cell-intrinsic mechanism linking immunoproteasome dysfunction to dysregulated polyamine metabolism in maturing neurons. Pharmacologic inhibition of ODC with DFMO partially rescues metabolic and neuronal differentiation phenotypes, supporting polyamine dysregulation as a therapeutically targetable metabolic pathway underlying CNS pathology in CANDLE/PRAAS. More broadly, these findings establish a conceptual link between proteasome function and metabolic regulation of neurodevelopment, with potential relevance to other proteasome- and polyamine-associated neurodevelopmental disorders, suggesting shared stress-response programs beyond monogenic autoinflammatory syndromes.

## Methods

### Sex as a biological variable.

Our study included both male and female patients, as described in Supplemental Tables 1 and 2. We did not observe any clear sex-related differences in this study.

### IQ testing.

Cognitive function was assessed with age-appropriate standardized tests: Wechsler Adult Intelligence Scale 4^th^ edition or Wechsler Intelligence Scale for Children 5^th^ Edition. Whole IQ scores are reported in Figure 1. Patient intelligence quotient (IQ) testing was compared to age-matched norms reflected in standardized scores for each test.

### Virus culture.

La Crosse virus (LACV) stocks derived from a 1978 human case were generated as previously described (59). Plaque forming units (PFU) of virus stocks were determined by plating 10-fold serial dilutions onto Vero cells (ATCC# CCL-81). Cells were cultured in 2% FBS and penicillin/streptomycin (Gibco) containing DMEM (Gibco) media. Plates were incubated for 1h to allow virus attachment and then each well was overlayed with 500mL of 1.5% carboxymethyl cellulose (Sigma) in MEM (Gibco). Plates were incubated five days, then fixed with 4% formaldehyde. Plates were then rinsed with de-ionized water and stained with 0.35% crystal violet. Plates were rinsed, air dried, and the numbers of plaques counted. Titers were calculated as the number of plaques per 1mL of supernatant applied.

### iPSC culture.

iPSCs were generated from fibroblasts isolated from patient skin biopsies (60). The HC1 iPSC line was obtained commercially from ATCC (https://www.atcc.org/products/acs-1023). HC2 iPSCs has been previously described as being generated from a skin punch biopsy and reprogrammed using ReproRNA -OKSGM (61). iPSCs were maintained in reduced growth factor Matrigel (Corning)-coated 25cm^2^ culture flasks in mTeSR Plus media (StemCell Technologies) with frequent culture splitting (~every 5 days) to maintain ≤70% confluency to minimize cellular differentiation. Splitting involved detaching cells with enzyme-free cGMP ReLeSR (StemCell Technologies) for ≤3min to leave differentiated cells attached to the flask and transferring replicating iPSCs to a new flask. iPSCs were fed daily with mTeSR Plus to maintain high levels of fibroblast growth factor in the culture to inhibit differentiation.

### NPC and mature neuron generation.

NPC and mature neuron cultures were generated using the STEMdiff SMADi Neural Induction kit’s monolayer culture protocol (StemCell Technologies). Cells were initially plated at 2×10^5^ cells/cm^2^. After passage 3 (18–21 days post induction), cells were transferred into Neural Progenitor Medium. For further neuronal differentiations, cells were plated at 1×10^5^ cells/cm^2^ at passage 3 of NPC differentiation in STEMdiff SMADi Neural Induction Medium. After 24 hours, the media was removed and replaced with STEMdiff Forebrain Neuron Differentiation Medium, which was changed daily for 6 days. Then cells were replated at 5×10^4^ cells/cm^2^ in STEMdiff Forebrain Neuron Maturation Medium and cultured for a minimum of 14 days to allow maturation. Medium was changed every three days or as required.

### Cerebral organoid generation, culture and drug administration.

COs were generated from CANDLE and HC iPSCs using the CO differentiation kit (StemCell Technologies) as described in Lancaster and Knoblich (62). Specifically, 5000 iPSCs were plated into each well of a 96-well plate with 50μl of Organoid Formation Media containing 5μM dihydrochloride and cultured for 5 days with an additional 50μl of media (without dihydrochloride) added on days 2 and 4. On day 5, embryoid bodies (EBs) were transferred to a new 96 well plate containing 150μL of neural induction media per well for a further 2 days. The start of neural induction (day 5) represents the “birthday” of each organoid. On day 7 (2-days old), neural-induced EBs were embedded into 20μL of Matrigel (Corning) which was polymerized at 37°C for 30min, then transferred to 2-3mL of Expansion media per 10-15 EBs in a 6 well plate. On day 10 (5-days old), Matrigel-embedded organoids were transferred to a 125mL Erlenmeyer flask with 25mL of Maturation media for an additional 30 days (25-days old for metabolic analysis) or 31 days (26-days old for immunohistochemistry). Media was replaced every 4-5 days. For metabolic analysis, organoids at day 30 were transferred to individual wells in 1 mL of Maturation media, in a 24 well plate for 24 hrs. prior to tissue and supernatant collection and processing on day 31 (26 days old). Organoids used for immunohistochemistry were fixed in 10% neutral buffered formalin on day 31 (26 days old) for further processing.

For DFMO treatment of COs, DFMO was dissolved directly in maturation media at 100mM stock concentration, and dilutions made with maturation media. COs were transferred to individual wells within a 24 well plate on day 11 (6-days old) and 1mL of control or maturation media containing DFMO was added to each well. Media was refreshed daily for each organoid until day 31 (26-days old) when tissue and supernatant was collected for either metabolic or transcriptomic analysis.

### Organoid infection.

COs were infected as previously described (27). Maturation media containing either 10^3^ PFU of LACV, INKV or mock Vero cell supernatant per 1mL of media were added to individual COs at 21 days post- neural induction for 2h with shaking at 85rpm in the incubator. 1mL of COs were washed with fresh media to remove virus and 1mL of fresh media added. COs were incubated for 6 days post infection (dpi), with culture supernatants being collected at 2, 4 and 6 dpi. At 6dpi, uninfected mock control, INKV and LACV COs had whole RNA collected as described below in the RNA isolation section. Uninfected mock controls were used as a baseline measurement of IFN responses in LACV and INKV infected COs.

### Organoid viability assay.

Organoids treated with normal maturation media or different concentrations of DFMO were analyzed for cellular reductive capacity using a PrestoBlue assay (ThermoFisher). This is a resazurin-based assay that serves as an indicator of cellular viability. Following maturation media removal, individual organoids were incubated for 30min with 250μL of 1XPrestoBlue diluted into maturation media from a 10X stock. 50μL of each sample was read in triplicate in a flat-bottom 96 well plate (Corning) using fluorescence excitation at 560nm and emission at 590nm on an BioTek Synergy 4 (Agilent) plate reader. 1X PrestoBlue media incubated without an organoid was used as a media blank control. Baseline measurements for each organoid were taken 24hr prior to drug treatment and then readings were taken every 3 days following DFMO administration out to 21 days post treatment for toxicity studies (Supplemental Figure 5, A and B) or 15 days for metabolic and transcriptomic analysis (Supplemental Figure 5, C and D). Data are reported as percent 590nm fluorescence relative to a pretreatment baseline.

### Bioplex assay.

Analysis of cytokine/chemokine concentrations was performed on undiluted supernatant from uninfected mock controls, INKV and LACV infected CO cultures using the Bio-Plex Human Inflammation Panel 37-plex (BioRad) per the manufacturer’s protocol. All data was collected on a Luminex 200 Instrument System (Luminex) and concentrations in range of detection were determined by Standard Curve analysis in Microsoft Excel.

### Metabolomics sample extraction.

For all liquid chromatography tandem mass spectrometry (LC-MS/MS) methods, LCMS grade solvents were used. All CO and biofluid samples were directly immersed in 0.4 mL of ice-cold methanol. NPCs and neurons in culture were first washed with 1 mL of 0.9 % NaCl, prior to the addition of 0.4 mL ice-cold methanol. Cells were then scraped to suspend all debris and transferred to microtube for further processing. To each sample 0.4 mL of water and 0.4 mL of chloroform were added. Samples were shaken for 30 minutes under refrigeration and centrifuged at 16k ×g for 20 min. 400 μL of the top (aqueous) layer was collected and diluted as necessary in 50 % methanol in water for LC-MS/MS analysis.

### Metabolomics LCMS.

Tributylamine was purchased from Millipore Sigma. LCMS grade water, methanol, isopropanol, formic acid, and acetic acid were purchased through Fisher Scientific. Aqueous metabolites were analyzed using two analytical methods with opposing ionization polarities (63, 64). Both methodologies utilized a LD40 XR UHPLC (Shimadzu Co.) system for separation and a 6500+ QTrap mass spectrometer (AB Sciex Pte. Ltd.) for detection. In negative mode methodology, samples were separated with a Waters Atlantis T3 column (100Å, 3 μm, 3 mm × 100 mm) and eluted using a binary gradient from 5 mM tributylamine, 5 mM acetic acid in 2% isopropanol, 5% methanol, 93% water (v/v) to 100% isopropanol over 5 minutes. Two distinct multiple reaction monitoring (MRM) ion pairs in negative mode were used for each metabolite where available. In positive mode methodology, samples were separated using a Phenomenex Kinetex F5 column (100 Å, 2.6 μm, 100 x 2.1 mm) and eluted with a gradient from 0.1 % formic acid in water to 0.1 % formic acid in acetonitrile over 5 minutes.

### Biocrates targeted metabolomics assay.

A targeted metabolomic assessment was carried out following the manufacturer’s protocol using Biocrates’ MxP Quant 500 kit (www.biocrates.com; Biocrates, Innsbruck, Austria). Six organoids, 300 μl of 85% Ethanol and 15% PBS were placed in 2 ml tubes from the Precellys Lysing Kit, soft tissue homogenizing CK14 (Bertin, Rockville, MD). The samples were then homogenized for 2 cycles of 20 s at 6500 rpm with 30 s intervals between homogenization steps using the Precellys 24 at 0–4°C. Afterwards, the samples were centrifuged in a bench top centrifuge, the solution transferred to new 1.5 ml Eppendorf tubes, and then centrifuged for 10 mins at 17,000-x g in a cold room. Samples were stored at –80^o^C until analysis.

The Biocrates MxP 500 was run using a Nexera HPLC system (Shimadzu) coupled to a 6500_+_ QTRAP mass spectrometer (AB Sciex) with an electrospray ionization source as previously described (13). Data were quantified using WebIDQ software and normalized to internal quality controls. Any metabolite that had greater than 30% above limit of detection (LOD) was excluded, while those with less than 30% LOD had their values imputed as 1/5 the lowest concentration.

### Immunoblotting and analysis.

iPSC-derived NPCs or neurons from HC1, HC2, CAN1, CAN2 and/or CAN3 were plated into a 12 well plate at 5x105 cells per well. Lysates from whole COs from the same iPSC sources were used for the same analysis. Lysates were generated from individual wells of NPCs or neurons by addition of cold 250μL of RIPA buffer (ThermoFisher) with 1X Halt Protease Inhibitor (ThermoFisher) for 5min followed by gentle trituration of the solution. CO lysates were generated in 500μL of the same lysis buffer with the same procedure. Lysates were collected in tubes on ice and then spun at 15,000g for 15min to remove debris. For ODC blots, a Peirce BCA analysis was performed on all samples and 5μg of total protein was loaded per well into a Criterion XT 10% Bis/Tris acrylamide gel (BioRad). For PSMB8 blots, 25μl of original RIPA lysate was loaded in each lane to maximize protein content for each sample. Proteins were electrophoretically separated by SDS-PAGE at 200V for 50min then transferred to an Immun-Blot PVDF (BioRad) membrane via wet transfer in 1X Tris-Glycine at 100V for 1hr. The membrane was rinsed twice in tris-buffered saline (TBS) for 10min and blocked in blocking buffer (TBS with 5% milk (BioRad) and 1% TritonX100 (Sigma-Aldrich)) for 1hr at room temperature (RT) with 80rpm shaking. Primary antibodies against ODC (Abcam, 1μg/mL), PSMB8 (Cell Signaling Technologies, 1:1000) or GAPDH (Novus, 5ng/mL) diluted in blocking buffer were applied overnight at 4°C with 80rpm shaking. The membrane was rinsed 3x5 min in TBS-T and an alkaline phosphatase-conjugated or horseradish peroxidase-conjugated secondary (Goat α Rabbit or Goat α mouse, 1:1000) was applied for 2hr at RT with 80 rpm shaking. The blot was them rinsed 3x15min in TBS-T and AttoPhos Substrate (Promega) or SuperSignal West Femto Maximum Sensitivity Substrate (ThermoFisher) was applied for 5min. Blot was visualized using the chemiluminescent setting on an iBright Imaging System (ThermoFischer).

### IHC.

At 26 days old, COs were placed in 10% neutral buffered formalin for 3-7days. COs were rinsed 3 × 1hr in PBS, then incubated at 30% sucrose in 1XPBS at 4°C overnight. Organoids were embedded into OTC compound (Sakura) contained within a 15x15mm cryomold (EMS) and frozen on block dry ice. 10μm sections were generated on a Leica CM3050S cryostat (Leica Biosystems) and maintained at -20°C until used. Sections were warmed to RT, residual dry OTC removed, and outlined with a Dako hydrophobic barrier pen (Agilent) for on-slide immunolabeling. Sections were washed 3x5min with PBS and blocked for 30min at RT with 5% normal donkey serum (Millipore Sigma). Rabbit anti-Sox2 (Abcam, 1:1000) or mouse anti-Tuj1 (B3T, Millipore Sigma, 1:500) primary antibody was applied in blocking buffer overnight at 4°C. Sections were washed 3x5min in PBS and AlexaFluor secondary antibodies donkey anti-Rabbit 488 or donkey anti-mouse 594 (ThermoFisher, 1:1000) in PBS were applied for 2hrs at RT. Sections were washed once with PBS and Hoechst 33342 (ThermoFisher, 0.1μg/mL) dissolved in PBS was applied for 15min. Sections were washed 3x5min in PBS and slides were coverslipped with Prolong Diamond antifade mountant (ThermoFisher) and #1.5 cover glass (Ted Pella). Sections were images in their entirety using a Leica Versa Slide Scanner (Leica) with an HC Plan APO 20x objective (NA 0.75).

### RNA isolation and quantitative reverse transcription (qRT) PCR from Cos.

At 26 days old, COs were collected in a new tube and media removed. COs were dissociated by adding 0.5mL of Corning Cell Recovery Solution (Corning) to each tube and incubating for 1hr on ice with intermittent (~every 15min) gentle trituration. Dissociated tissues were washed with PBS 3 times by pelleting the sample at 500g and replacing with fresh supernatant. 600μL of ZR RNA buffer (Zymo) was added to the sample and gentle trituration was used to dissolve the cell pellet. Whole RNA was isolated and according to the manufacturer’s Quick-RNA Midiprep kit protocol with the exception that the DNAse digestion was performed at 37C for 30min using Ambion DNAse I (RNase-free) reagent. RNA concentrations were determined spectrophotometrically using a Nanodrop One (ThermoFisher) and RNA (100ng) was reverse transcribed to cDNA using an iScript reverse transcription kit (Bio-Rad) per the manufacturer’s instructions. Reactions were performed on a MasterCycler nexus GX2 (Eppendorf) using a 5 min priming at 25°C, 30 min RT reaction at 42°C, a 5 min RT inactivation at 85°C and a 20 min stabilization at 20°C. cDNA was diluted 5-fold in nuclease-free water prior to us real-time PCR analysis.

Primers to detect human *GAPDH*, *SOX2*, *TUBB3*, *SSAT, IFNβ1, IFIT1, IFIT2, ISG15, MAP2,* and *NEUN* (Supplemental Table 4) were designed using the NCBI primer design tool (NCBI) and confirmed to be specific for the target gene by blasting against the NBCI database. Primer sequences are shown in the Key Resources Table. All primers were tested on cDNA derived from normal organoids and confirmed to amplify a single target by dissociative melt curve analysis. SYBR Green dye with ROX (Bio-Rad) was used for all real-time PCR analysis per the manufacture’s protocol. Samples were run in a 384 plate in triplicate on a QuantStudio Pro7 Real-time PCR system. No reverse transcriptase and water only controls were used. Data from each sample was calculated as the percent difference in Cq value with the housekeeping gene *Gapdh* (ΔCq=(Cq *Gaph*)- (Cq gene of interest)).

### Flow cytometry.

At 26 days old, HC1 and CAN1 COs were dissociated with Cell Recovery Solution as described above. A single cell suspension was generated by passing the dissociation solution through a 70μm filter (Corning). In a 5mL tube, fixable viability dye eFluro 780 (Invitrogen, 1:1000) in PBS with 0.04% BSA was applied for 30min on ice, in the dark to all cells except an unlabeled control aliquot. Cells were then distributed into a 96 well plate for further processing, including wells designated for unlabeled, single channel, and fluorescence-minus-one controls. Cells were washed 2x by pelleting at 500g for 3min, removing the solution by flicking the plate and resuspending cells in PBS with BSA. Cells were then fixed, permeabilized and labeled with primary conjugate antibodies against Sox2 (Biolegend, 1:50) and Tuj1 (Abcam, 1:125) with the eBioscience Foxp3/Transcription Factor Staining Buffer Set as indicated by the manufacturer. Briefly, cells were incubated in fixation/permeabilization solution overnight at 4°C and then permeabilization buffer with Fc receptor blocking Truestain FcX (Biolegend, 1:1000) was added for 15min on ice in the dark. Cells were spun and flicked and then permeabilization buffer containing primary antibodies was added to designated wells for 30min on ice in the dark. Cells were washed 1x with permeabilization buffer and then 2x with1X PBS by spinning and flicking. After the final wash, cells were resuspended in 200μl of PBS and analyzed by a BD FACSymphony (BD Biosciences). Gating layout is shown in Supplemental Figure 1.

### Analysis of immunohistochemistry performed on COs.

Immunohistochemistry using Sox2 and Tuj1 was performed on sections from HC1 (*n* = 15), HC2 (*n* = 19), CAN1 (*n* = 22), HC2 (*n* = 19), and HC3 (*n* = 12) individual organoids for the initial IHC experiments. A total of 229 sections from HC COs and 426 sections from CANDLE COs were imaged using the same capture settings for each label on the Leica Aperio slide scanner as described above. For the control versus DFMO experiments, 12 organoids were used per iPSC line (HC1, HC2, CAN1, CAN2, CAN3) per treatment, except for CAN2-DFMO, which had 10 organoids analyzed, with 2-3 sections per organoid for a total of 342 sections analyzed. Both label channels were captured in the same imaging session into a single RGB image. Using ImageJ analysis software (NIH), the Sox2, Tuj1 and Hoechst 33342 channels were split using the “Split Channels” function into individual images, converted to 8-bit gray scale images and saved. Using the Hoechst channel image, a freehand selected ROI was generated for each imaged organoid and saved to be applied to the associated single channel images to define the tissue area. Tissue with excessively damaged or large regions devoid of Hoescht label was removed from the analysis. Each 8-bit single channel image was made binary under the process tab to generate a positive label mask. The ROI generated for the Hoechst label was then overlayed onto the Sox2 or Tuj1 masked image to define tissue area. Analyze Particles function was then used with the following settings to define the percent of CO tissue area positive for each label: Size (pixel^2) =1-Infinity, Circularity=0.00-1.00, Show=Masks, with the “Display results” and “Summarize” tabs selected. Numbers of positive cells were completed in a blinded study, without knowing the information of each image being analyzed, and then compiled afterwards.

### Ratio metric analysis of protein bands in immunoblots.

Grayscale images of whole immunoblots were captured on an iBright 1500 and inverted for ratio metric analysis. In Fiji, a region of interest (ROI) was generated to contain the entirety of the largest target protein band on the blot, but as little unlabeled area as possible. That same ROI was used to measure all other bands on the blot as well as background subtract regions. An integrated density measurement was taken for each band of the protein of interest (ODC or PSMB8) and GAPDH loading control. A blank area on the blot immediately adjacent to each band was also measured to be used as a background subtract. Background subtracted density measurements of the target protein for each sample were divided by the background subtracted density of GAPDH of the same sample were plotted.

### Analysis of LCMS-derived metabolic data.

All signals were visually inspected for presence and integrated using SciexOS 3.1 (AB Sciex Pte. Ltd.). Metabolites with multiple MRMs were quantified with the higher signal to noise MRM. Filtered datasets of the negative mode aqueous metabolites were total sum normalized after initial filtering. The positive mode aqueous metabolomics dataset was scaled and combined with the negative mode aqueous metabolite dataset using signals for metabolites common to both methods including Arginine, Tyrosine and Threonine. Goodness of the scaling was assessed by Principle Components Analysis. A Benjamini-Hochberg method for correction for multiple comparisons was imposed where indicated in text or Figure captions. Technical powering was used across donors for CO-related metabolomics statistics.

### Statistics.

Statistical analysis was completed based on the most relevant test for individual experiments, as described in figure legends. A 2-tailed unpaired students *t* test, a 2-tailed 1-way ANOVA and a 2-tailed 2 way ANOVA were used for statistical analysis. *P* values of less than 0.05 were considered significant. Statistical analysis was done utilizing Graph Pad Prism software, with the exception of metabolomic data whose analysis is described above in “Analysis of LCMS-derived metabolic data”.

### Study approval.

All research investigations were done as part of natural history protocol 17-I-0016/NCT02974595 and as such were approved by the NIAID Institutional Review Boards. The patients were referred to the NIH between 2007 and 2013. All patients and their parents provided consent or assent for study enrollment and the study procedures including IQ testing, skin biopsies, iPSC generation and lumbar punctures. The study was reported according to CARE guidelines and conducted in compliance with the Declaration of Helsinki principles.

### Data availability.

Dataset information for all metabolomic studies are available at: https://data.mendeley.com/datasets/k7kf9cym4g/1 Other data generated are included in this published article and Supplemental figures or are available from the corresponding author upon request. Values for all data points in graphs are reported in the Supporting Data Values file.

## Author contributions

CWW and BS contributed equally to the data generation, analysis, and writing and are listed as cofirst authors. CWW initiated the project and is listed first. CWW, BS, CLH, RGM, and KEP were involved in the experimental design, data analysis, and writing of the manuscript. CWW, BS, KW, SA, STF, JS, LJ, AT, CLF, GC, EB, FB, NTB, RM, CDK, and CLH generated data. MB, GC, BB, JK, and PK generated and/or procured samples including iPSCs. All authors provided valuable feedback and reviewed the manuscript.

## Conflict of interest

The authors have declared that no conflict of interest exists.

## Funding support

- The Intramural Research Program of the National Institutes of Health (NIH) through AI001102-11 (KEP), AI001220-9 (RGM) and AG000297 (NIA/RM).

## Supplementary Material

Supplemental data

Unedited blot and gel images

Supporting data values

## Figures and Tables

**Figure 1:**
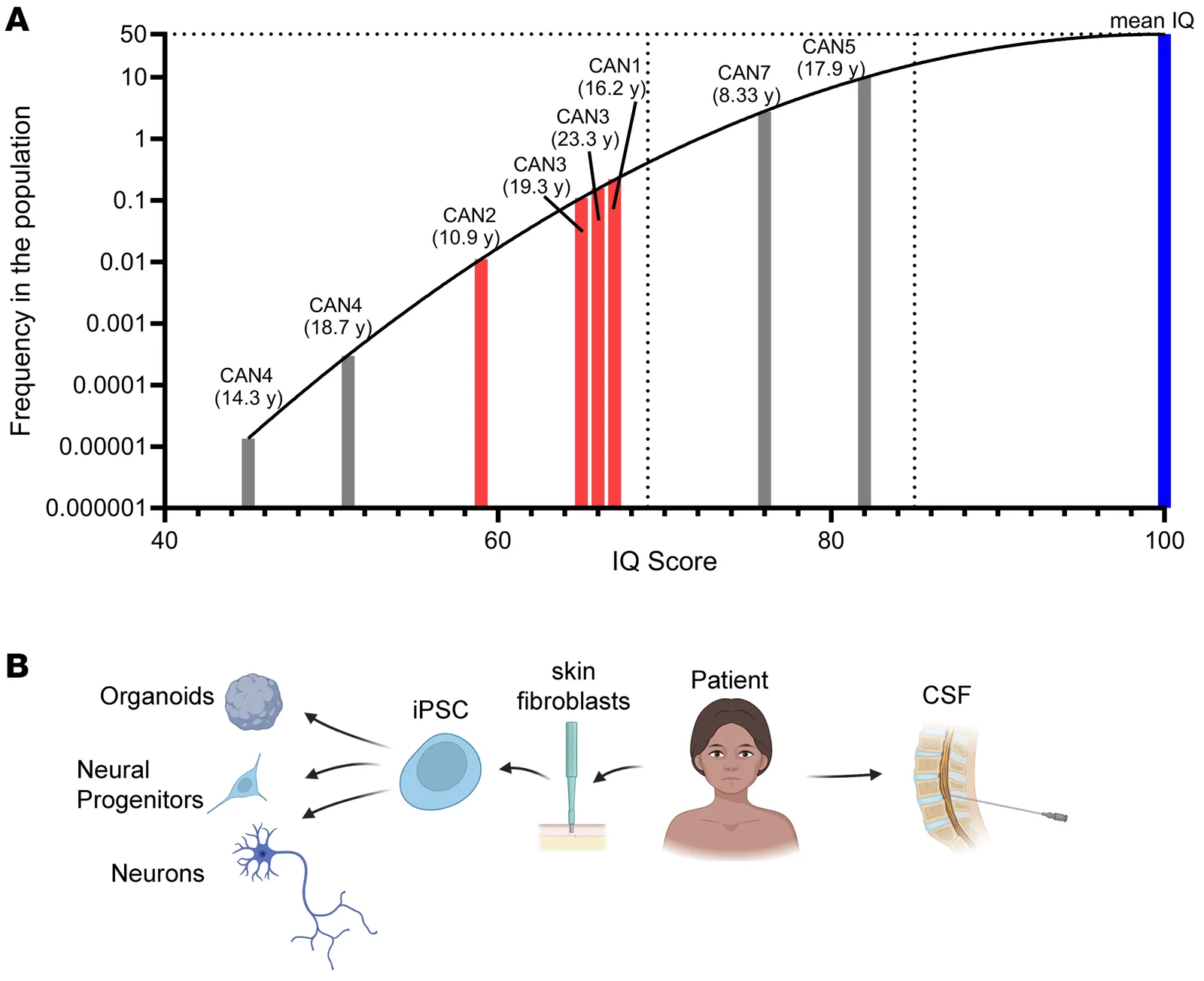
Patients with CANDLE have lower than average Wechsler Intelligence scores. (**A**) The plotted black line represents the frequency (y-axis) of an IQ score (x-axis) in a normal population with the blue bar to the right indicating an IQ of 100 (the mean standardized IQ score, SD = 15). Age-appropriate Wechsler Intelligence Scale testing results of 3 CANDLE patients, for which iPSCs were generated were plotted as the red bars. Patients where iPSCs were not generated are plotted as gray bars. The age at each testing is given above the bar. The horizontal dotted line indicates a population frequency of 50 and the 2 dotted vertical lines indicate an IQ of 69 (intellectual disability) and 85 (low normal) from left to right. The results demonstrate low IQ scores for all patients, implying impaired neurodevelopment. (**B**) Schematic showing sample collection, processing, tissue generation, and analysis from patients with CANDLE. For patients CAN1, CAN2, and CAN3, skin biopsies were used to generate iPSCs from skin fibroblasts. These iPSCs were then utilized to generate cerebral organoids, neural precursor cells, and neurons. CSF was used from patients CAN3, CAN6, and CAN7 for metabolomic analysis. There was no IQ score for CAN6.

**Figure 2:**
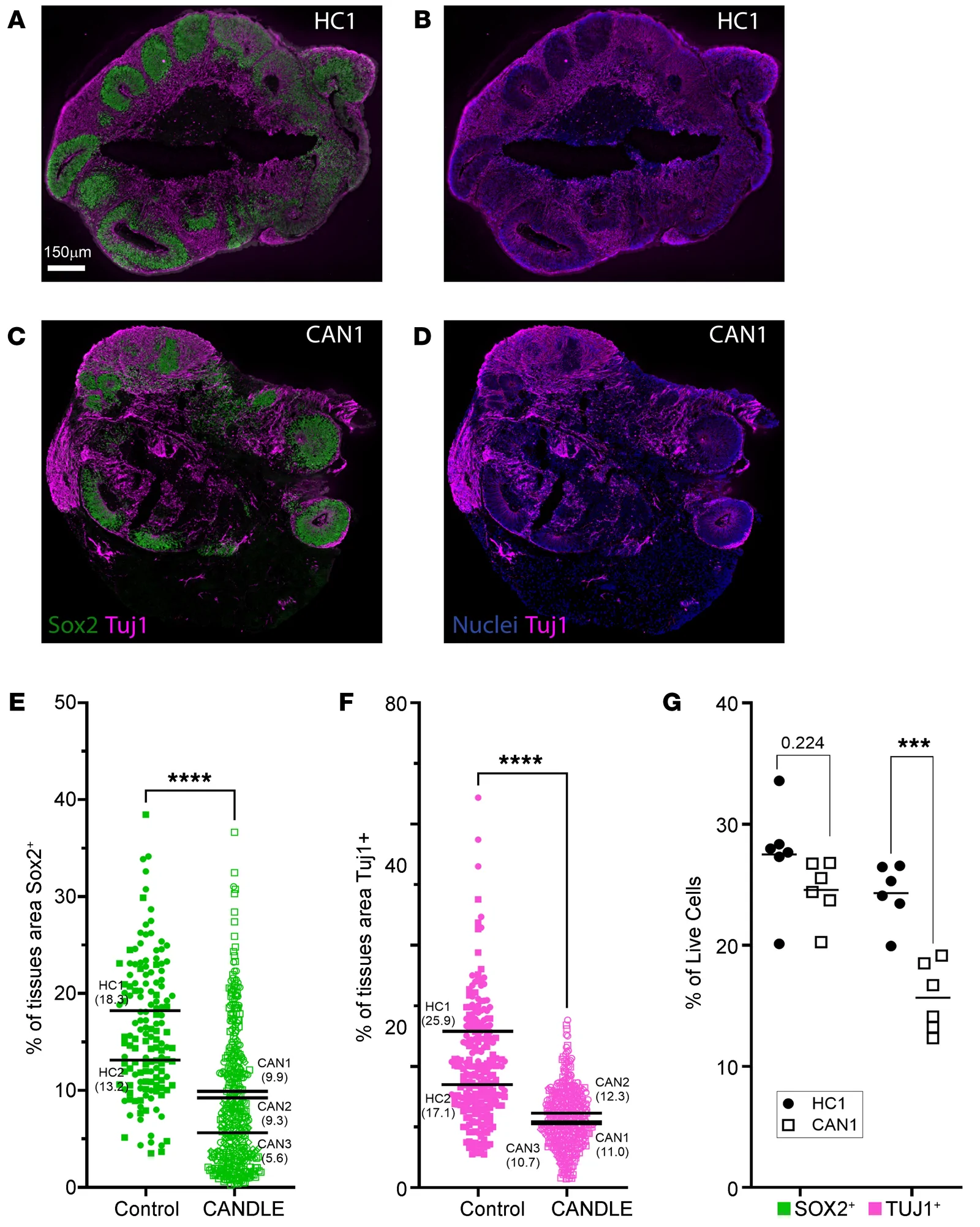
CANDLE COs have altered development. (**A**–**F**) IHC analysis of neural progenitor (SOX2) and mature neuron (TUJ1) markers in (**A** and **B**) HC1 and (**C** and **D**) CAN1 cerebral organoids. Blue signal is cell nuclei. Quantification of IHC labeling of (**E**) SOX2 and (**F**) TUJ1 analyzed on sections from *n* = 56 CANDLE and *n* = 31 healthy control organoids. Data are plotted as percent of each signal within each tissue section using symbols described in Supplemental Table 1 to indicate COs generated from different iPSCs. An unpaired *t* test between each group was performed. ****P < 0.0001. **(G)** Flow cytometry was performed on *n* = 6 organoids generated from HC1 and CAN1 with labeling for the same SOX2 and TUJ1 markers. Data is shown as the percentage of positive live cells from whole dissociated organoid. A 2-way ANOVA with multiple comparisons was performed. ***P < 0.001. Other *P* values are indicated with specific values.

**Figure 3:**
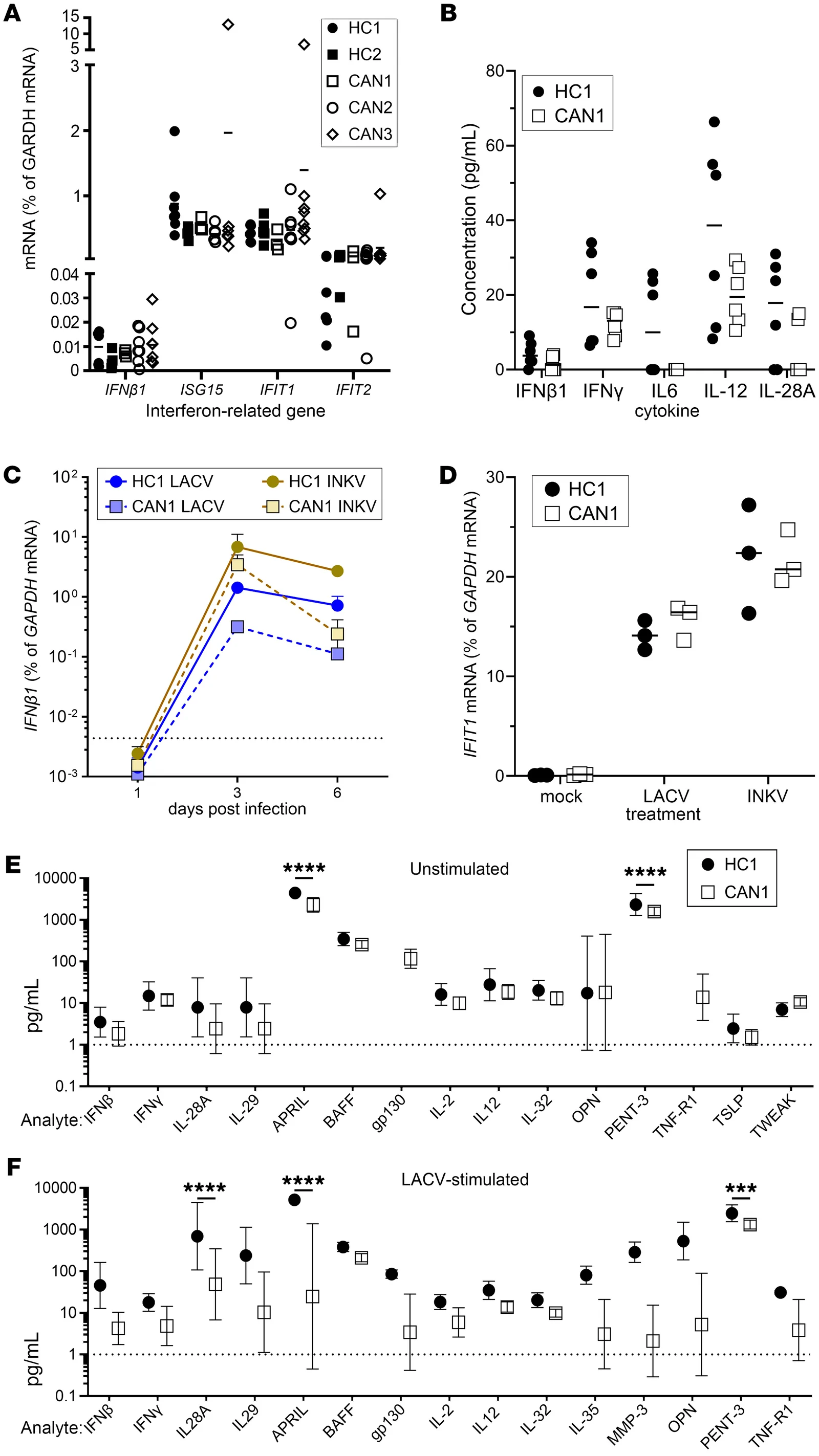
CANDLE COs do not have elevated cytokine or IFN responses. (**A**) cDNA reverse transcribed from mRNA isolated from COs from HC1, HC2, CAN1, CAN2, and CAN3 was analyzed by real-time PCR for the indicated genes. Data, as analyzed as the ratio of *GAPDH* mRNA expression relative to the gene of interest for each organoid. A 2-way ANOVA with a Šidák’s multiple comparisons test was used to compare these group. No statistical difference was observed. (**B**) Supernatants from HC1 and CAN1 COs at 3 weeks of age were measured for the concentration of cytokines related to the IFN response using a human inflammation 37 bio-plex multiplex immunoassay. An unpaired *t* test between HC and CANDLE was used to compare groups. No statistical difference was observed. (**C** and **D**) HC1 and CAN1 COs were infected with 10^3^ PFU of La Crosse virus (LACV) or Inkoo virus (INKV). At the indicated time points, COs were collected and mRNA extracted and reverse transcribed. Individual COs were analyzed for (**C**) *IFNB1* and (**D**) *IFIT1* mRNA expression. For all graphs, each symbol represents data from individual organoids or supernatants with 2–8 samples per group per time point. An unpaired *t* test was used to compare HC and CANDLE within the mock, LACV, and INKV groups. No significance was observed. (**E** and **F**) Averages of analytes from the human inflammation 37 bioplex assay (also shown as individual points in **B**) for (**E**) uninfected and (**F**) LACV-infected COs (*n* = 6 for all groups) Data are shown for analytes that were consistently detectable above background for either mock or infected samples. Data are shown as the geometric mean ± geometric SD. A 2-way ANOVA with a Šidák’s multiple comparisons test was used to compare groups. ***P < 0.001, ****P < 0.0001.

**Figure 4:**
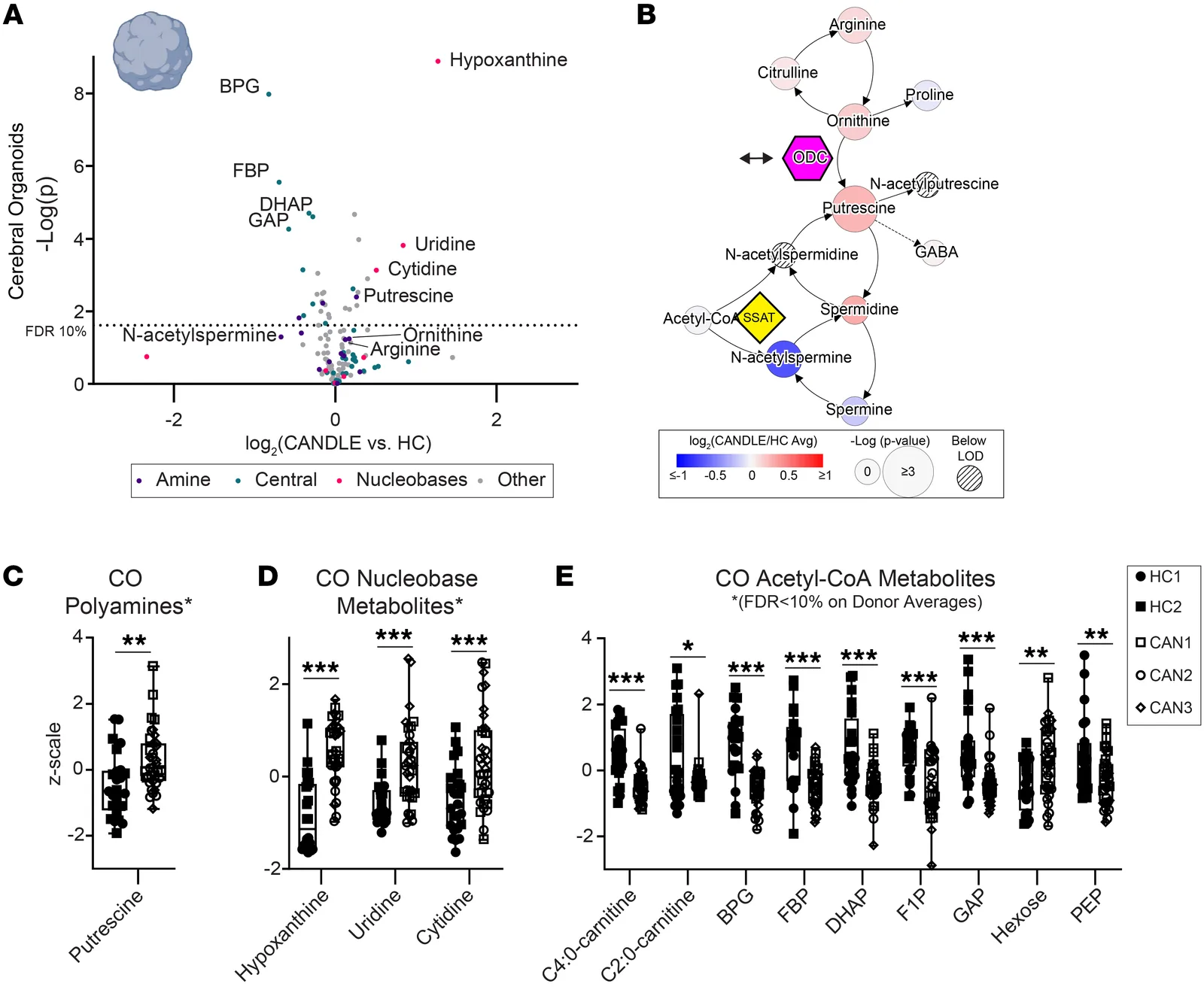
COs from patients with CANDLE have altered polyamine, nucleobase, and acetyl-CoA metabolism. (**A**) A comparison of CANDLE versus HC CO metabolites via *t* test (technical: *n* = 12 per donor, biological: *n* = 3 CANDLE, *n* = 2 HC). Metabolites annotated as being associated with amine metabolism dark blue dots), Acetyl-CoA metabolism (light blue dots), and nucleobase metabolism (red dots) are indicated. A line representing the raw *P* value cut off for an FDR of 10 % calculated via a Benjamini-Hochberg correction is indicated. (**B**) Amine metabolism map of the same CO metabolite comparison displayed in **A** with the fold change of metabolites represented as color and the significance represented as node size as indicated in the legend. Hatch marked nodes indicate metabolites below the limit of detection. Yellow symbols indicate enzymes involved in regulating key processes, ODC for the conversion of ornithine to putrescine and SSAT for the inactivation of polyamines via acetylation. CO expression of the key (**C**) polyamine putrescine, (**D**) nucleobases and (**E**) Acetyl-CoA metabolites that passed an FDR 10 % cutoff by Benjamini-Hochberg correction. Data are shown for all assayed COs in z-scale using the variance of all samples to normalize the distribution for comparison purposes. Range, quartiles, and median are shown as box and whiskers. An unpaired *t* test was used to compare between groups *P < 0.05, **P < 0.01, ***P < 0.001.

**Figure 5:**
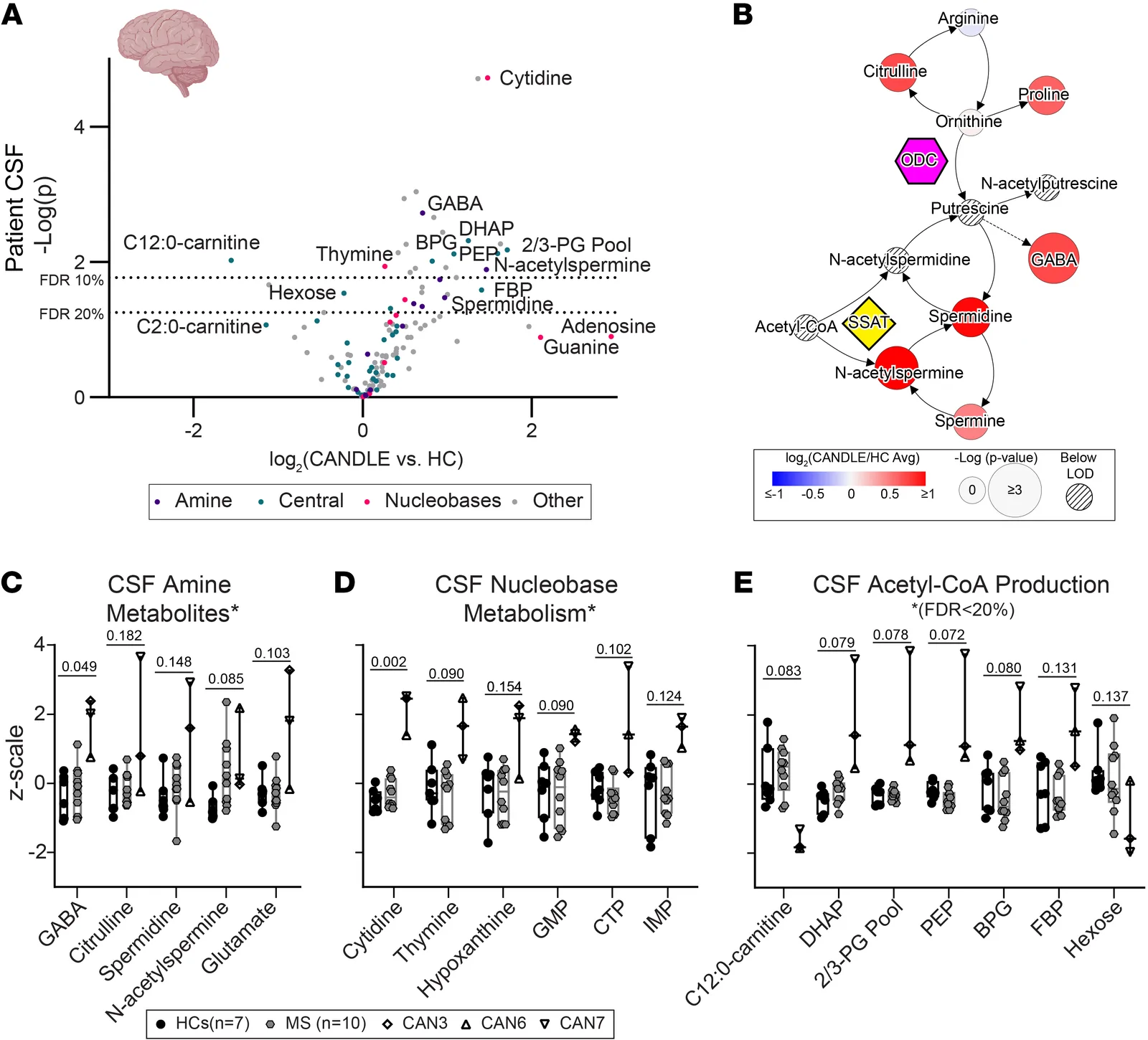
CSF from patients with CANDLE have altered polyamine, nucleobase, and Acetyl-CoA metabolism. (**A**) A comparison of CANDLE versus HC CSF metabolites via *t* test (biological: *n* = 3 CANDLE, *n* = 7 HC). Metabolites annotated as being associated with amine metabolism, Acetyl-CoA metabolism, and nucleobase metabolism are highlighted. Lines representing the raw *P* value cutoff for an FDR of 10 % or 20 % calculated via a Benjamini Hochberg correction are indicated. (**B**) Amine metabolism map of the same metabolite data and comparison displayed in **A** with the fold change of metabolites represented as color and the significance represented as node size as indicated in the legend. Hatch marked nodes indicate metabolites below the limit of detection. Patient CSF data for (**C**) amine, (**D**) nucleobase, and (**E**) Acetyl-CoA metabolites that passed an FDR 20 % cutoff are shown for each individual patient or control. Data are plotted in z-scale using the variance of all samples to normalize the distribution for comparison purposes. The range, quartiles, and median are indicated by box and whiskers. An unpaired *t* test was used to compare between CANDLE and HC and the Benjamini-Hochberg corrected *P* value is displayed above each comparison.

**Figure 6:**
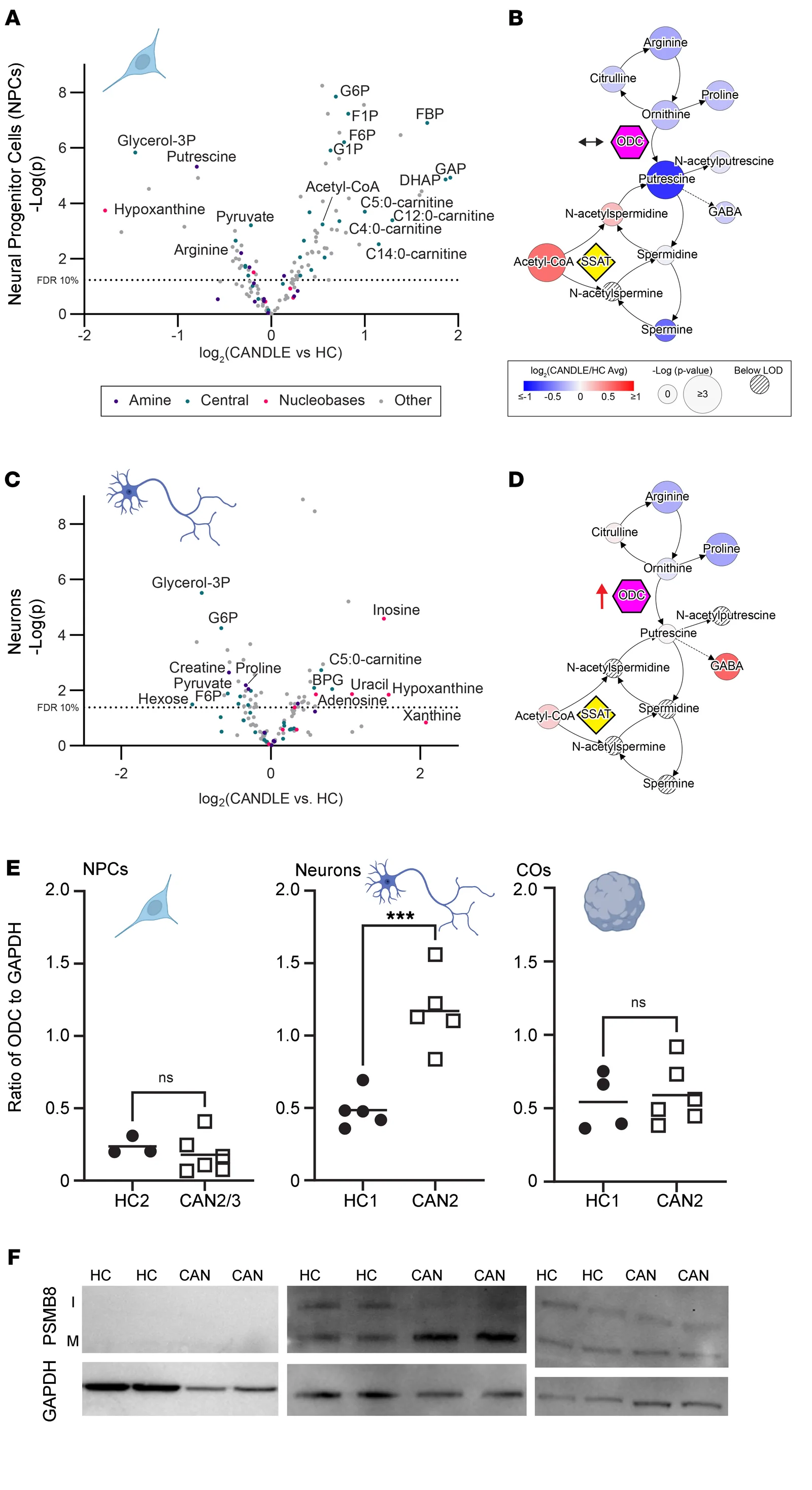
NPCs and Neurons with CANDLE mutation have altered polyamine metabolism. (**A**) Comparison of CANDLE and HC NPC metabolites via *t* test (technical *n* = 6, biological: *n* = 2 CANDLE, *n* = 3 HC). Metabolites annotated as being associated with amine metabolism (purple dots), Acetyl-CoA metabolism (light blue dots), and nucleobase metabolism (magenta dots) are indicated. A line representing the raw *P* value cutoff for an FDR or 10 % calculated via a Benjamini-Hochberg correction is indicated. (**B**) Amine metabolism map of the same NPC data displayed in **A** with the fold change of metabolites represented as color and the significance represented as node size, as indicated in the associated legend. Hatch marked nodes indicate metabolites below the limit of detection. (**C**) Comparison of CANDLE and HC Neuron metabolites via *t* test (technical: *n* = 6, biological: *n* = 3 CANDLE, *n* = 3 HC). Metabolites annotated as being associated with amine metabolism (purple dots), Acetyl-CoA metabolism (light blue dots), and nucleobase metabolism (magenta dots) are indicated. (**D**) Amine metabolism map of the same neuron metabolite data displayed in **C** with the fold change of metabolites represented as color and the significance represented as node size. (**E**) Ratio metric densitometry analysis of Western blot banding of ODC protein relative to GAPDH in CANDLE versus HC iPSC-derived NPCs (left graph), neurons (middle graph), and COs (right graph). Each point represents an individual lysate generated from a single well of a confluent 12 well plate of either NPCs or neurons or from a single CO. The donor source of the iPSC-derived cultures or COs are indicated in the graph. Data were compared via a 2-tail, unpaired *t* test. ***P < 0.001. (**F**) Representative Western blot expressed of PSMB8 protein by iPSC-derived NPCs (left graph), neurons (middle graph), and COs (right graph). The immature form of PSMB8 (**I**) is approximately 26 kD in molecular weight and the mature form is approximately 21 kD. The same blots were reprobed for GAPDH expression at approximately 39 KD (bottom blots). Lysates from iPSC derived NPCs (HC1, HC2, CAN1, and CAN3) neurons (HC1, CAN1, CAN2, and CAN3) and COs (HC1, HC2, CAN1, CAN2, and CAN3) were all examined. Banding patterns between HC and CAN were found to be similar within each sample type. Specifically, NPCs had little-to-no PSMB8 expression in either genotypic group while neurons and COs had prominent bands present in all samples. Whole blots with all samples tested are shown in the supplement.

**Figure 7:**
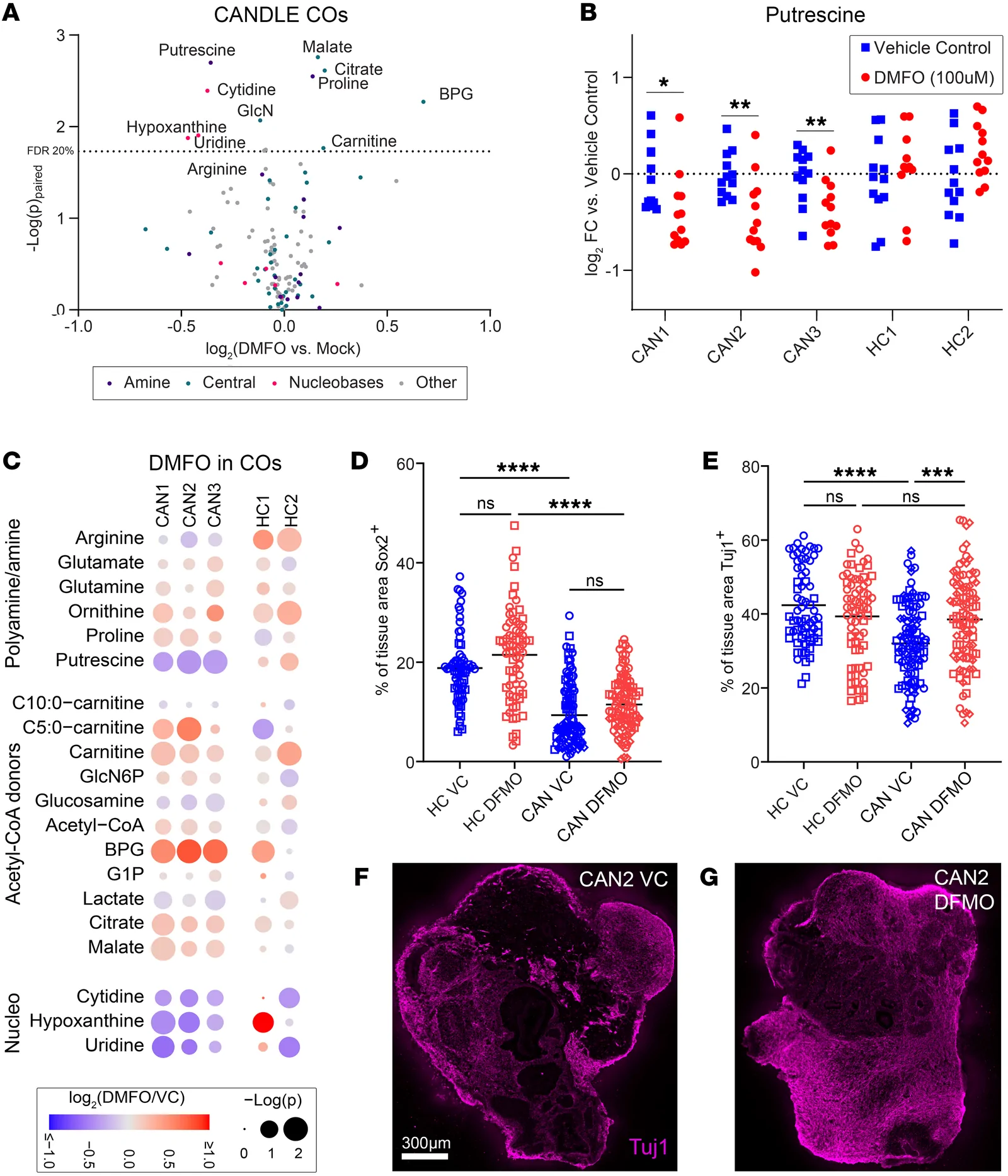
Impact of DFMO on metabolism and neuronal development in CANDLE COs. (**A**) Comparison of 100 μM DFMO-treated CANDLE COs versus vehicle-treated CANDLE CO metabolites via paired *t* test by on the average metabolite levels (CO technical *n* = 12, biological *n* = 3 CAN). Metabolites annotated as being associated with amine metabolism (purple dots), Acetyl-CoA metabolism (light blue dots), and nucleobase metabolism (magenta dots) are indicated. (**B**) Change in putrescine signal in COs relative to the average of the vehicle control (VC) by donor. An unpaired *t* test was used to compare groups. *P < 0.05, **P < 0.01). (**C**) Metabolites that changed in response to DFMO treatment in CANDLE COs via a paired *t* test with *P* < 0.1 organized by pathway. Node color reflects the fold change due to DFMO and the size reflects the significance of that change by patient as indicated in the associated legend. (**D** and **E**) Quantification of IHC labeling of SOX2 and TUJ1 analyzed on sections from *n* = 10–12 vehicle-treated and *n* = 12 DFMO-treated CANDLE COs. Data are plotted as percent of each signal within each tissue section using symbols described in Supplemental Table 1 to indicate COs generated from different iPSCs. Data in both **D** and **E** were analyzed by a 2-way ANOVA with Šidák’s multiple comparisons test. *P < 0.05, ****P < 0.0001. Representative IHC labeling of the mature neuron marker TUJ1 in **F** VC-treated and (**G**) DFMO-treated CAN2 COs.

**Table 1:**
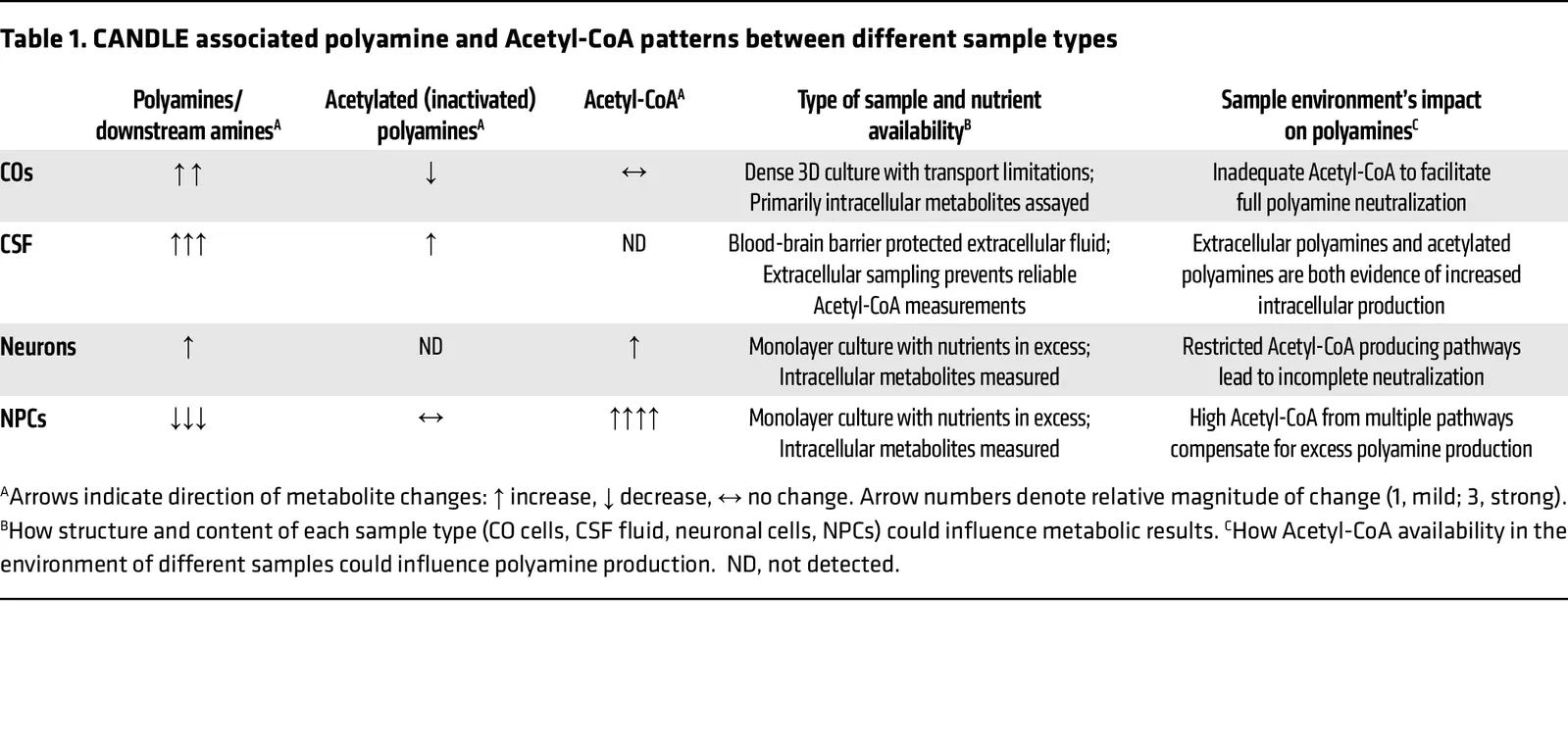
CANDLE associated polyamine and Acetyl-CoA patterns between different sample types

## Version 1 08/04/2026

In-Press Preview

## Version 2 10/01/2026

Electronic publication
