# Supplementary material for: Proteasome mutations associated with CANDLE syndrome cause altered neuronal development by dysregulating polyamine synthesis
Source: J Clin Invest. 2026 Aug 4;136(19):e199140. doi: 10.1172/JCI199140 (PMC13626833; doi:10.1172/JCI199140)
Supplement: Supplementary file 1 [file jci-136-199140-s269.pdf]

Supplemental Material for:

Proteasome mutations associated with CANDLE syndrome cause altered neuronal development by dysregulating polyamine synthesis

Clayton W. Winkler[1*], Benjamin Schwarz[2*], Katie Williams[3], Sara Alehashemi[4], Simote T. Foliaki[3], Joseph Snow[5], Lisa Joseph[6], Audrey Thurm[6] Christopher Friend[4], Gwendolyn Cooper[2], Eric Bohrnsen[2], Farzana Bhuyan[4], Nathan T. Brandes[2], Ruin Moaddel[7], Manfred Boehm[8], Guibin Chen[8], Cole D. Kimzey[2], Bibiana Bielekova[9], Joanna Kocot[9], Peter Kosa[9], Cathryn L. Haigh[3], Raphaela Goldbach-Mansky[4#], Karin E. Peterson[1#]

[1] Neuroimmunology Section, Laboratory of Neurological Infections and Immunity (LNII), Rocky Mountain Laboratories (RML), National Institute of Allergy and Infectious Diseases (NIAID), NIH, Hamilton, MT, USA,
[2] Research and Technologies Branch, RML, NIAID, NIH, Hamilton, MT USA
[3] Prion Cell Biology Unit, LNII, RML, NIAID, NIH, Hamilton, MT USA
[4] Translational Autoinflammatory Diseases Section, Laboratory of Clinical Immunology & Microbiology (LCIM), NIAID, NIH, Bethesda, MD, USA
[5] Neuropsychology Consult Service, Intramural Research Program, National Institute of Mental Health (NIMH), NIH, Bethesda, MD, USA
[6] Neurodevelopmental and Behavioral Phenotyping Service, Intramural Research Program, NIMH, NIH, Bethesda, MD, USA
[7] Laboratory of Clinical Investigation, Biomedical Research Center, National Institute on Aging, NIH, Baltimore, MD USA
[8] Laboratory of Cardiovascular Regenerative Medicine, Translational Vascular Medicine Branch, National Heart, Lung, and Blood Institute, NIH, Bethesda, MD, USA
[9] Neuroimmunological Diseases Section, LCIM, NIAID, NIH, Bethesda, MD, USA

* These authors contributed equally to this project and share first authorship
# These authors contributed equally to this project and share senior authorship

Raphaela Goldbach-Mansky,
Bldg 10, Clinical Center,
9000 Rockville Pike10 Center Dr, Bethesda, MD 20892
phone: 301-761-7553
e-mail: goldbacr@mail.nih.gov

Karin E. Peterson
Rocky Mountain Laboratories
903 S. 4th St., Hamilton, MT 59840
Phone: 406-375-9630
Email: petersonka@niaid.nih.gov

Conflict of interest statement: The authors have declared that no conflict of interest exists

Table S1. Derivation of iPSCs used for organoid generation.

ID[a]	*Diagnosis*[b]	*Mutation(s)*[c]	*iPSC source*[d]	*Source Ref*[e]	*Reprog*[f]	*Add Info*[g]	*Age*[h]	*Sex*	*symb*[i]
CAN1	CANDLE	PSMB8(T75M)/ PSMA3 digenic	patient skin fibroblast		Episomal Vectors	ADHD	11.8	M	□
CAN2	CANDLE	PSMB8 (T75M)	patient skin fibroblast		Episomal Vectors	-	12.5	F	○
CAN3	CANDLE	PSMB8 (T75M/ T92A)	patient skin fibroblast		Sendai virus	-	24.8	M	◇
HC1	Healthy	-	skin fibroblast	ATCC (1023)	Retroviral	-	36	F	●
HC2	Healthy	-	skin fibroblast	RAH019A	Plasmid	-	67	F	■
HC3	Healthy	-	skin fibroblast	ASE-9209	Episomal	-	47	F	◆

a: identification of patient for this study

b: diagnosis of patient.

c: mutation associated with CANDLE

d: source of cells for generation of iPSCs

e: source reference for control iPSC lines

f: how cells were reprogrammed for iPSCs

g: additional information of patients. ADHD: Attention-deficit/hyperactivity disorder; NK, normal karyotype

h: Age at which iPSCs were generated

i: symbol used to reference this patient/iPSC/CO throughout the manuscript

Table S2. Age and Sex information for CSF samples

ID	AGE	SEX	GENOTYPE
**HC4**	29	M	
**HC5**	50	M	
**HC6**	43	F	
**HC7**	54	F	
**HC8**	50	F	
**HC9**	46	M	
**HC10**	41	F	
**CAN3**	20	M	compound heterozygous *PSMB8*
**CAN6**	3.5	M	digenic *PSMB4, PSMB9*
**CAN7**	7.5	F	*PSMB8* dominant negative
**MS1**	27	F	
**MS2**	74	M	
**MS3**	34	F	
**MS4**	47	F	
**MS5**	58	F	
**MS6**	58	F	
**MS7**	28	M	
**MS8**	59	M	
**MS9**	49	M	
**MS10**	67	M	

Supplemental Figure 1

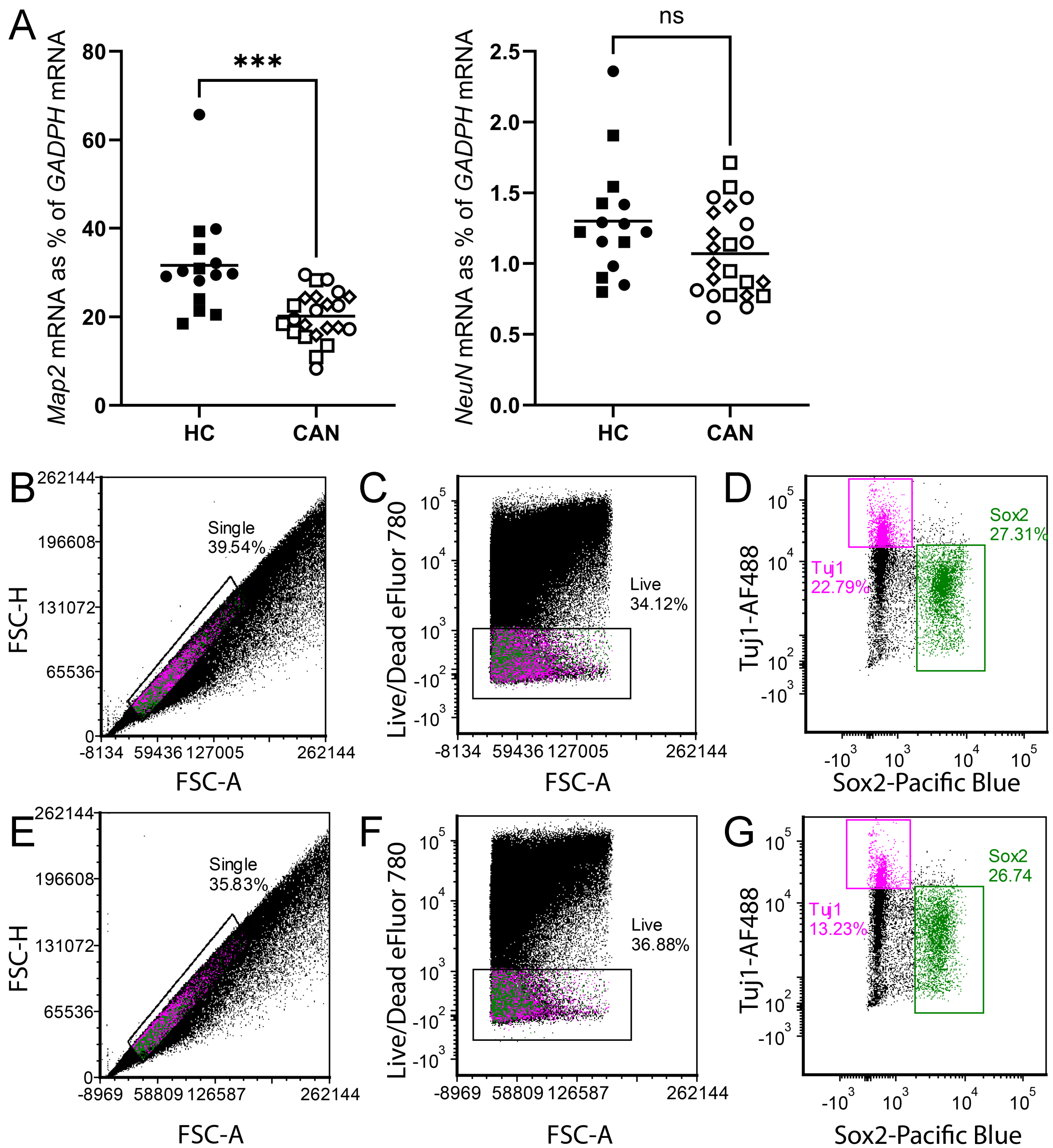

Figure S1. Gene expression and flow cytometric gating strategy and analysis of cerebral organoids. Transcriptional expression of MAP2 and NEUN from n=16 healthy control and N=24 CANDLE COs. Data are plotted as transcription expression as a percentage of GAPDH housekeeping gene relative to the mean of healthy control percent of GAPDH. Data was analyzed by a student's T test. *** indicates p<0.001 (A). Flow cytometry was performed on n=6 dissociated COs from HC1 (B-D) and CAN1 (E-G). Doubles were removed from analysis by including events outside the diagonal when plotting FSC height x area (B&E). Live cells were identified by excluding labeling events labeled with Live/Dead eFluor 780 (C, F). Within the live cell population, SOX2 (green) and TUJ1 (magenta) immunolabeling was quantified (D&G).

Supplemental Figure 2

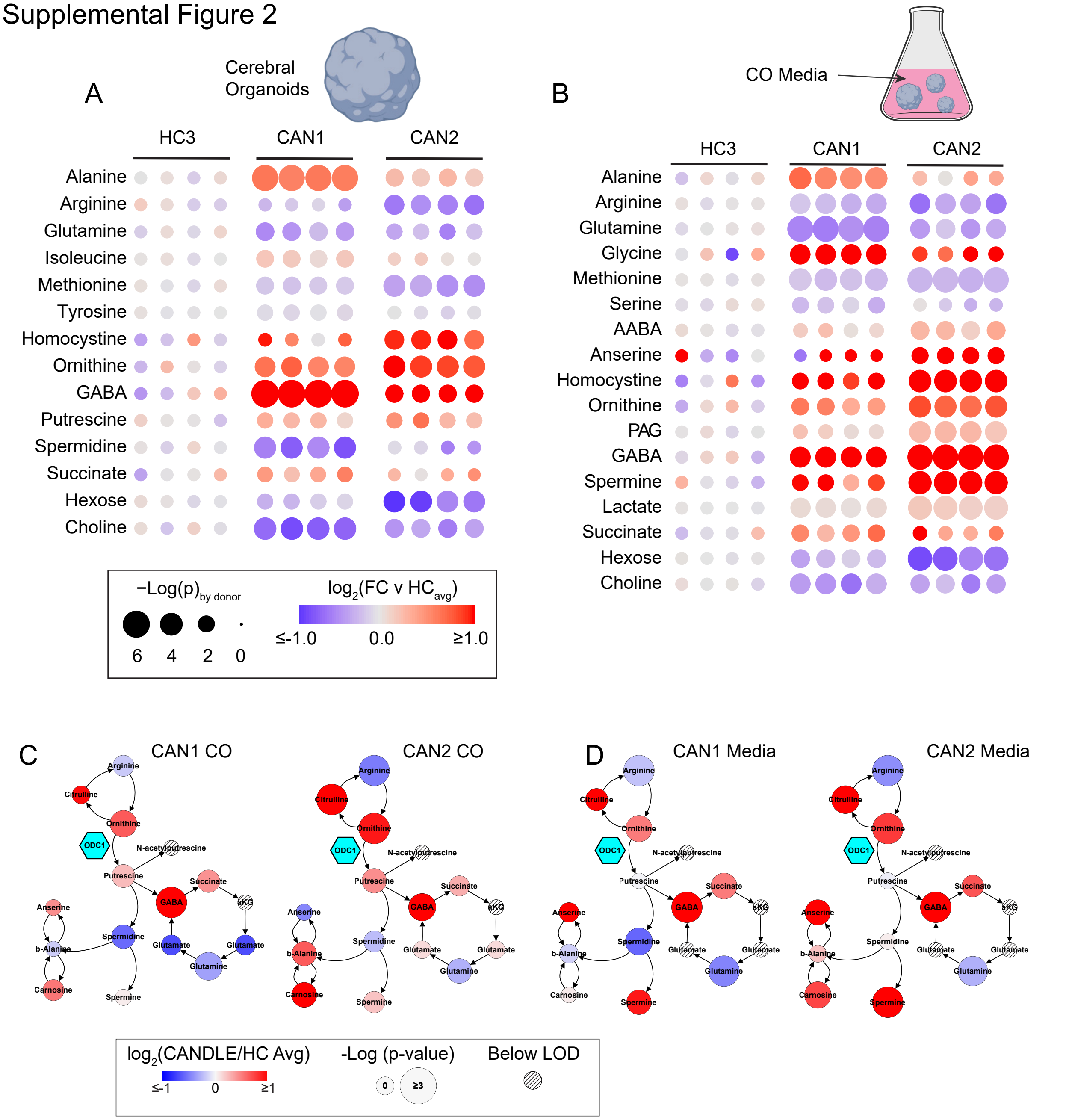

Figure S2. Biocrates metabolomics for COs and CO media. A Biocrates Quant500 kit was used to measure metabolites from (A) COs and (B) CO media from HC3, CAN1, and CAN2. Displayed metabolites significantly varied between HC and CAN, with CAN1 and CAN2 pooled. Significance defined as passage of a 10 % FDR correction for multiple comparisons. In A&B the color of each node reflects the individual fold change (as the base two logarithm) for each CO analyzed compared to the average value of the HC3 COs for that metabolite. The size of each node reflects the negative logarithm of the raw p-value from a comparison of that patient to HC3 by a t-test. Only non-lipid metabolites were included in this analysis. (C&D) Amine metabolism maps of the same CO and CO-media metabolite comparisons displayed in (A&B) with the fold change vs. HC3 represented as color and the significance represented as node size as indicated in the legend. Hatch marked nodes indicate metabolites below the limit of detection or not targeted via this assay.

Supplemental Figure 3

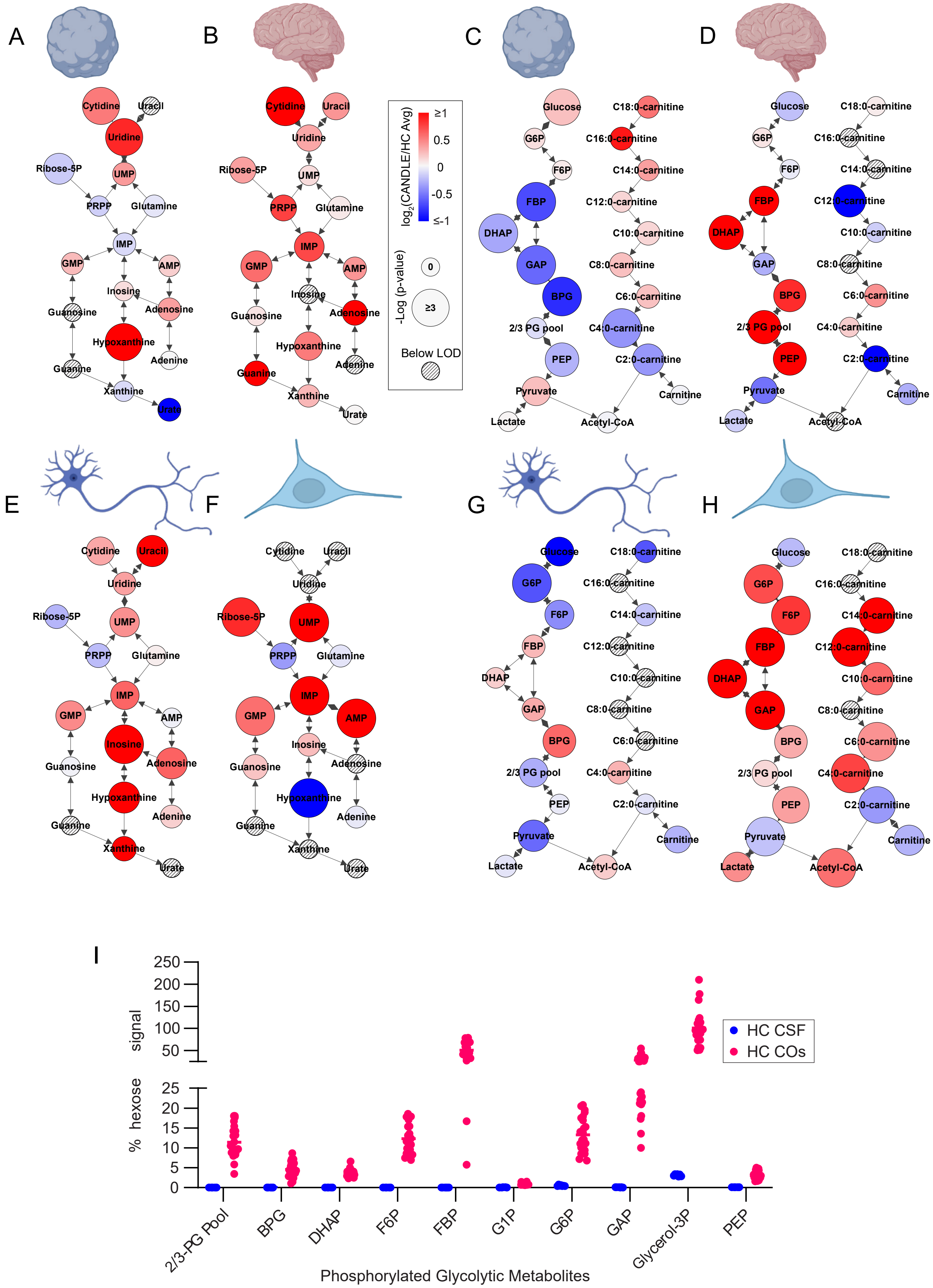

Figure S3. Cross matrix, CANDLE-driven nucleobase and acetyl-CoA directed metabolic patterns. (A&B) Metabolic maps of CANDLE-associated changes in metabolite profiles compared to HCs in nucleobase metabolism and nucleobase salvage from (A) COs and (B) CSF. (C&D) Metabolic maps of CANDLE-associated changes in metabolite profiles compared to HCs in Acetyl-CoA generating pathways from (C) COs and (D) CSF. (E&F) Metabolic maps of CANDLE-associated changes in metabolite profiles compared to HCs in nucleobase metabolism and nucleobase salvage from (E) iPSC-derived neurons and (F) iPSC-derived NPCs. (G&H) Metabolic maps of CANDLE-associated changes in metabolite profiles compared to HCs in Acetyl-CoA generating pathways from (G) iPSC-derived neurons and (H) iPSC-derived NPCs. Across all maps, fold change (as the base two logarithm) of metabolites in CANDLE samples compared the HC samples is represented as color and the raw p-value (as the negative logarithm) via a t-test between CANDLE and HC is represented as node size. Replicates by sample type were: COs technical n=12 per donor, biological n=3 CAN n=2 HC, CSF biological n=3 CAN n=7 HC, iPSC-derived neurons and NPCs technical n=6, biological n=3 CAN n=2 HC. (I) Comparison of signal-levels by LC-MS/MS of phosphorylated glycolytic intermediates by matrix in HC samples. All displayed metabolite signals were normalized to the signal for hexose as a related nonenergized metabolite.

Supplemental Figure 4

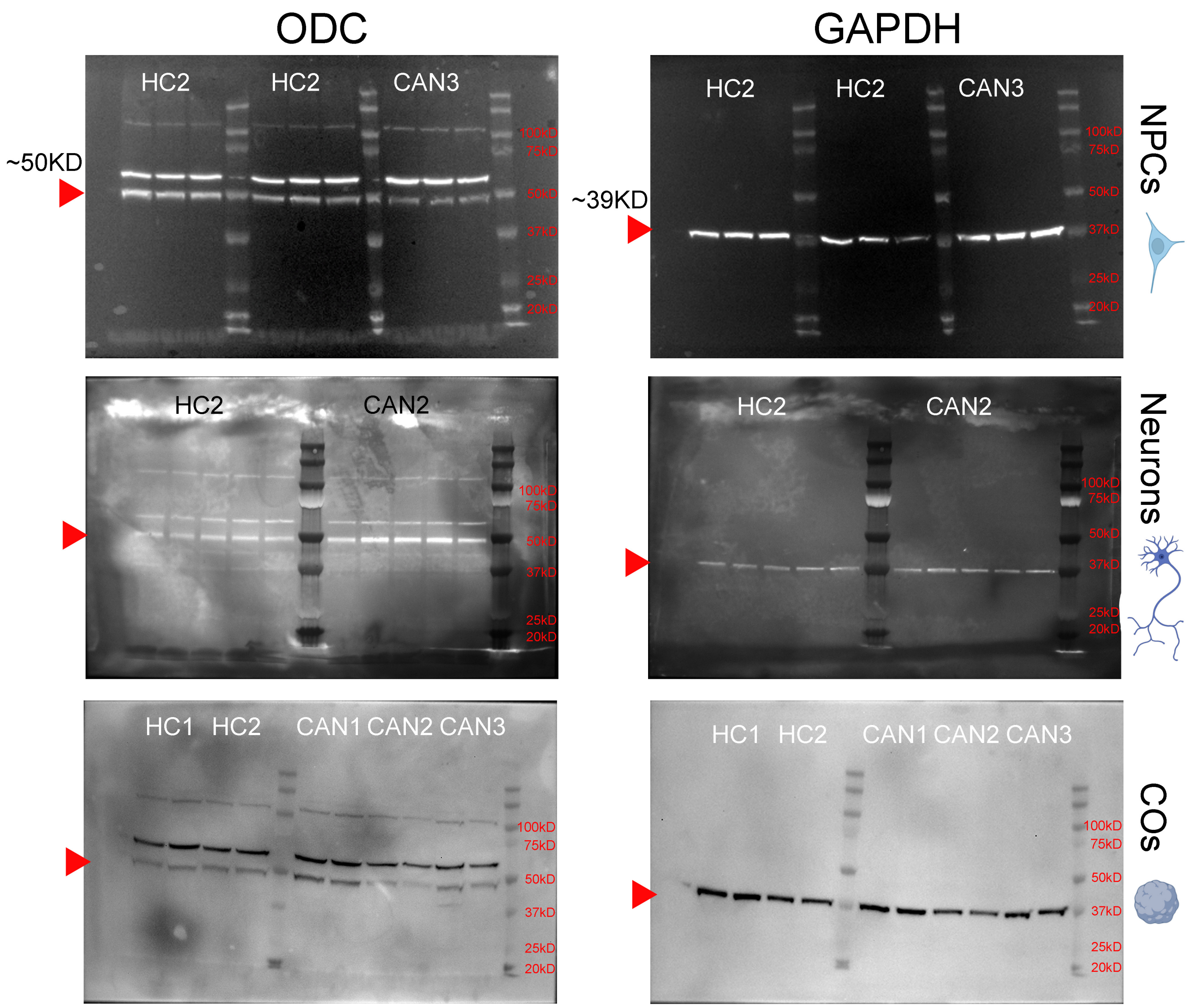

Figure S4. Whole western blots images of ODC expression. ODC expression in iPSC-derived NPCs (top left column), neurons (middle left column) and COs (bottom left column) generated from HC and CAN donor samples as indicated by the white labels associated with each lane. The monomeric ODC is ~50kD in molecular weight (red arrows, left column). The same blot was reprobed for GAPDH expression at ~39kD (right column). All blots were used to determine ODC to GAPDH ratios presented in Figure 6E.

Supplemental Figure 5

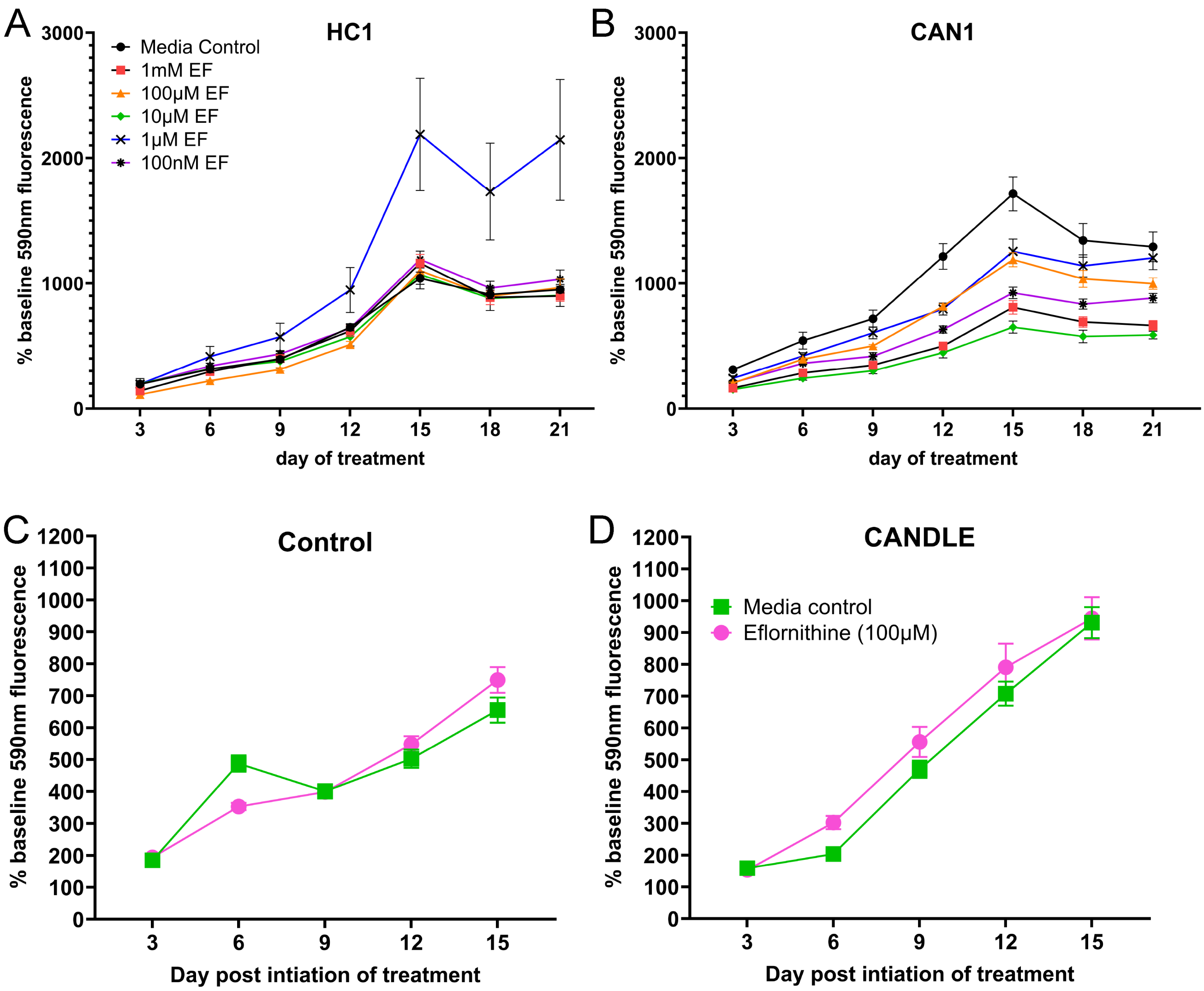

Figure S5. Toxicity assessment of DFMO in control and CANDLE organoids. COs generated from n=3 HC1 (A) and n=3 CAN1 (B) were administered daily varying concentrations of DFMO in the culture media to determine the drug's effect on cellular reductive capacity as an indicator of cell health. The highest DFMO concentration with a similar level in cellular reductive capacity to media control (100μM) was selected for further metabolic and transcriptional experiments. Metabolic capacity of n=16 COs generated from HC1 and HC2 (D) and n=24 COs (E) generated from CAN1-3 used in metabolic and transcriptional experiments (Figure 7) throughout treatment.

Supplemental Figure 6

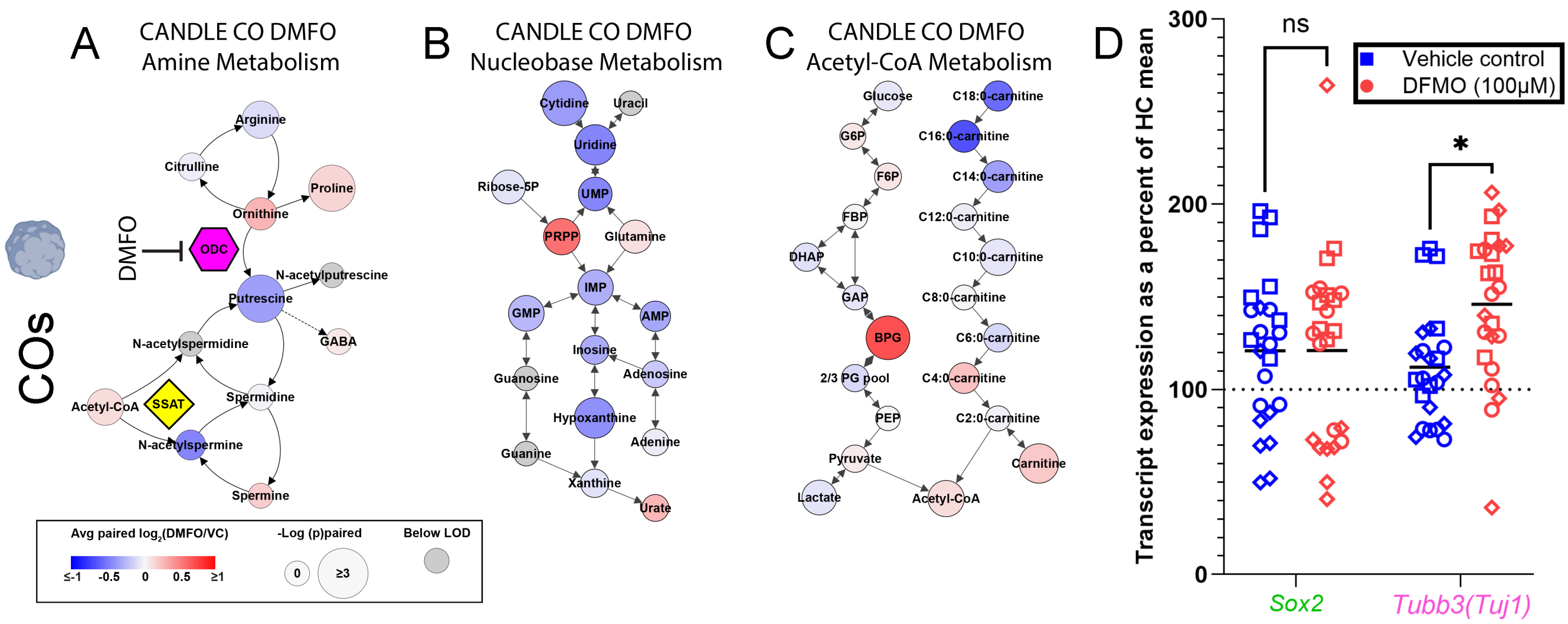

Figure S6. DFMO reverses CANDLE-associated amine, nucleobase, and Acetyl-CoA metabolic pattern in COs. (A) A map of changes in amine metabolism associated with the paired comparison of donor metabolite averages for 100 µM DMFO-treated CANDLE COs vs. vehicle controls with the fold change of metabolites represented as color and the significance represented as node size (CO technical n=12, biological n=3 CAN). (B) A map of DMFO driven changes in CANDLE COs in nucleobase metabolism using the criteria described in (A). (C) A map of DMFO driven changes in CANDLE COs in Acetyl-CoA generating pathways using the criteria described in A. (D) Transcriptional expression of SOX2 a and TUBB3 (Tuj1) with and without DFMO treatment from n=16 healthy control and N=24 CANDLE COs. Data are plotted as transcription expression as a percent of Gapdh housekeeping gene relative to the mean of healthy control percent of Gapdh. Data were analyzed by a 2way ANOVA with a Sidak's multiple comparisons test. * indicates $p<0.05$.

Table S3. Summerized interpretations of polyamine dysregulation patterns.

Metabolite / Node	Cerebral Organoids (CO)	Organoid Supernatant	CSF	NPCs	Neurons	Mechanistic Interpretation[b]
Ornithine[a]	↑↑↑	↑↑	↔	↓	↓	Amine metabolic disruption varies by model
Putrescine	↑↑	↔	↔	↓↓↓	↔	Intracellular putrescine accumulation dependent on model and availability of inactivation mechanisms
GABA	↑↑↑	↑↑	↑↑↑	↓	↑↑	Polyamine diversion toward GABA is a likely inactivation mechanism
Spermidine	↓↓	↓↓	↑↑↑	↔	ND	Marker of polyamine dysregulation *in vivo*
Spermine	↑	↑↑	↑↑	↓↓	ND	Marker of polyamine dysregulation across models
N-acetylspermidine	↔	ND	↔	↑	ND	Lack of effective spermidine-specific polyamine inactivation routes
N-acetylspermine	↓	↓	↑↑	ND	ND	Increased polyamine inactivation *in vivo*

a: Direction of metabolite changes: ↑ increase, ↓ decrease, ↔ no change, ND not detected (see Figures 4-6). Arrow numbers denote relative magnitude of change (1 =mild, 3= strong).

b: Proposed mechanism of polyamine dysregulation in COs, CSF and neurons. Increased ODC levels drive ornithine → putrescine, acetylated and nonacetylated polyamines in CSF and downstream GABA production in cerebral organoids (CO), CSF and neurons. NPC's had broader metabolic flexibility and did not show detectable PSMB8, suggesting alternative pathology.

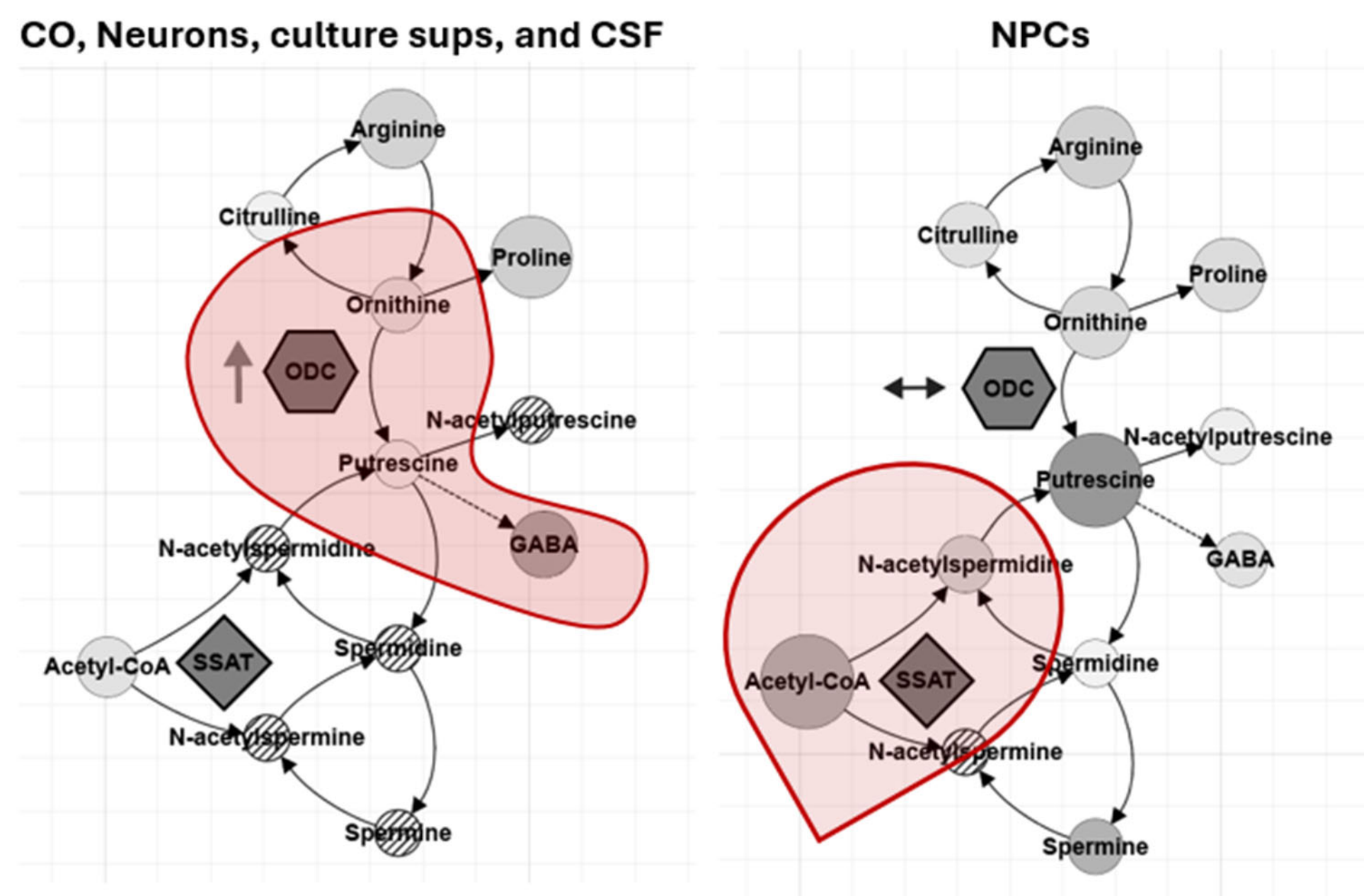

Table S4. Key Resource Table

REAGENT or RESOURCE	SOURCE	IDENTIFIER
Antibodies		
RabbitαODC1	Abcam	Cat# AB270268
RabbitαPSMB8/LMP7	Cell Signaling Tech	Cat#13635S
RabbitαSox2	Abcam	Cat# AB97959
Alexa Fluor® 488 MouseαTuj1	Abcam	Cat# 195879
Pacific Blue™ Mouseα Sox2	Biolegend	Cat# 656112
Human TruStain™ FcX (Fc Receptor block)	Biolegend	Cat# 4222302
MouseαTuj1 (B3T)	Millipore Sigma	Cat# MAB1637
MouseαGAPDH/G3PDH	Novus Biologicals	Cat# 686613
Alkaline Phosphatase GoatαRabbit	Jackson ImmunoReserach	Cat# 111-055-003
Alkaline Phosphatase GoatαMouse	Jackson ImmunoReserach	Cat# 115-055-003
Horseradish Peroxidase GoatαRabbit	Thermo Fisher	Cat# 65-6120
Horseradish Peroxidase GoatαMouse	Thermo Fisher	Cat# 62-6520
DonkeyαRabbit Alexa Fluor® 488	Thermo Fisher	Cat# A-21206
DonkeyαMouse Alexa Fluor® 594	Thermo Fisher Scientific	Cat#A-21203
Bacterial and virus strains		
La Crosse virus	Dr. Steven Whitehead (NIH)	Human 1978
Inkoo virus	Dr. Steven Whitehead (NIH)	SW AR 83-161
Chemicals, peptides and recombinant proteins		
Dako hydrophobic pen	Agilent	Cat# S2002
Immun-Blot PVDF membrane sandwhich	BioRad	Cat# 1620238
10% Criterion™ XT Bis-Tris Protein Gel, 12+2 well	BioRad	Cat# 3450111
Blotting Grade Blocker Non-Fat Dry Milk	BioRad	Cat# 1706404XTU
iTaq Universal SYBR Green Supermix	BioRad	Cat# 1725120
iScript cDNA synthesis kit	BioRad	Cat# 1708891
Growth factor reduced Matrigel	Corning	Cat# 354230
Matrigel® Matrix	Corning	Cat# 354234
Cell Recovery Solution	Corning	Cat# 354253
Tissue-Tek® Cryo-mold 15x15x15mm	EMS	Cat# 62534-15
DMEM	Gibco	Cat# 11995-065
MEM	Gibco	Cat# 12360-038
Penicillin-Streptomycin	Gibco	Cat# 15140122
eBioscience™ Fixable Viability Dye eFluor™ 780	Invitrogen	Cat# 65-0865-14
Ambion™ DNAse I (RNase-free)	Invitrogen	Cat# AM2222
Nuclease-Free Water	Invitrogen	Cat# AM9932

eBioscience™ Foxp3 / Transcription Factor Staining Buffer Set	Invitrogen	Cat# 00-5523-00
Tributylamine	Millipore Sigma	Cat# 139322500
Triton X-100	Millipore Sigma	Cat# X100-500ML
Donkey serum	Millipore Sigma	Cat# D9663-10mL
Bovine Serum Albumin	Millipore Sigma	Cat# A9647
AttoPhos® Substrate	Promega	Cat# S1012
SuperSignal™ West Femto Maximum Sensitivity Substrate	ThermoFisher	Cat# 34094
Tissue-Tek® O.C.T. Compound	Sarkura	Cat# 4583
Carboxymethylcellulose sodium salt, medium viscosity	Sigma	Cat# C4888
Formaldehyde Solution, 36.5-38% molecular biology grade	Sigma	Cat# F8775
Ethanol 95%	Sigma	Cat# 65348-M
Difluoromethylornithine (DFMO)	Sigma	Cat# 1234249
BrainPhys™	StemCell Technologies	Cat# 05790
STEMdiffTM SMADi Neural Induction kit	StemCell Technologies	Cat# 08581
STEMdiffTM Neural Progenitor Media	StemCell Technologies	Cat# 05833
STEMdiffTM Forebrain Neuron Differentiation Media	StemCell Technologies	Cat# 08600
STEMdiffTM Forebrain Neuron Maturation Medium	StemCell Technologies	Cat# 086056
mTeSR Plus media	StemCell Technologies	Cat# 100-1130
Enzyme-free cGMP ReLeSR	StemCell Technologies	Cat# 100-0438
Methanol Optima LCMS grade	Thermo Fisher	Cat# A4564
Water Optima LCMS grade	Thermo Fisher	Cat# W64
Isopropanol Optima LCMS grade	Thermo Fisher	Cat# 4614
Chloroform ACS grade	Thermo Fisher S	Cat# C606SK4
Acetic Acid Optima LCMS grade	Thermo Fisher	Cat# A11350
Formic Acid Optima LCMS grade	Thermo Fisher	Cat# 85178
Pierce BCA Protein assay kits	Thermo Fisher	Cat# 23225
Hoescht 33342	Thermo Fisher	Cat# 62249
PrestoBlue™ cell viability reagent	Thermo Fisher	Cat# A13261
RIPA lysis and extraction buffer	Thermo Fisher	Cat# 89901
Halt Protease inhibitor cocktail (100X)	Thermo Fisher	Cat# 78429
eBioscience Fixable viability eFluro™ 780 dye	Thermo Fisher	Cat# 65-0865-14
Prolong Diamond antifade mountant	Thermo Fisher	Cat# P36965
Quick-RNA Midiprep kit	Zymo Research	Cat# R1056
Critical commercial assays		
Bio-Plex Pro Human Inflammation Panel 37-plex	BioRad	Cat# 171AL001M

Deposited Data		
Metabolic datasets	Mendeley	https://data.mendeley.com/datasets/k7kf9cym4g/1
Experimental models: Cell lines		
Vero Cells	ATCC	Cat# CCL-81
KYOU-DXR0109B Human IPS cell ACS-1023	ATCC	Cat# 201B7
Oligonucleotides		
hGapdh.2 Forward primer	IDT	5′-TCGTGGAAGGACTCATGACC-3′
hGapdh.2 Reverse primer	IDT	5′-ATGATGTTCTGGAGAGCCCC-3′
hSox2.1 Forward primer	IDT	5’-GAGCTTTGCAGGAAGTTTGC-3’
hSox2.1 Reverse primer	IDT	5’-GCAAGAAGCCTCTCCTTGAA-3’
hTubb3.1 Forward primer	IDT	5’- CCAAGGGTCACTACACGGAG-3’
hTubb3.1 Reverse primer	IDT	5’-ATGATGCGGTCGGGATACTC-3’
hSSAT.1 Forward primer	IDT	5’-TACCACTGCCTGGTTGCAGAAG-3’
hSSAT.1 Reverse primer	IDT	5’- CTTGCCAATCCACGGGTCATAG-3’
hIFNb.1 Forward primer	IDT	5'-CTGCAACCTTTCGAAGCCTT-3'
hIFNb.1 Reverse primer	IDT	5'-AGAAGCACAACAGGAGAGCA-3'
hIFIT1.2 Forward primer	IDT	5'-GCTTTCAAATCCCTTCCGCT-3'
hIFIT1.2 Reverse primer	IDT	5'-GCCTTGGCCCGTTCATAATT-3'
hIFIT2.1 Forward primer	IDT	5'-CCGAACAGCTGAGAATTGCA-3'
hIFIT2.1 Reverse primer	IDT	5'-CCCTCCATCAAGTTCCAGGT-3'
hISG15.1 Forward primer	IDT	5'-TTGCCAGTACAGGAGCTTGT-3'
hISG15.1 Reverse primer	IDT	5'-GGGACACCTGGAATTCGTTG-3'
hMap2.1 Forward primer	IDT	5'-AGGCTGTAGCAGTCCTGAAAGG-3'
hMap2.1 Reverse primer	IDT	5'-CTTCCTCCACTGTGACAGTCTG-3'
hNeuN.3 Forward primer	IDT	5'-TGACCAACAAGAAGACGGGG-3'
hNeuN.3 Reverse primer	IDT	5'-AATTCAGGCCCGTAGACTGC-3'
Software and algorithms		
SciexOS (3.1.6.44)	AB Sciex	https://sciex.com/products/software/sciex-os-software
Cytoscape (3.10.3)	Cytoscape	https://cytoscape.org/
GraphPad Prism (10.2.0)		
Multiwell-System software (2.0.11.0)	MultiChannel Systems	https://www.multichannelsystems.com/downloads/software
NCBI primer design tool	NCBI	https://www.ncbi.nlm.nih.gov/tools/primer-blast/
ImageJ (v1.54n)	NIH	http://imagej.org
RStudio (R 4.3.3)	The R Project for Statistical Computing	https://www.r-project.org/
Other		
ReproRNA -OKSGM	StemCell Technologies	Cat# 05930

70mm cell strainer	Corning	Cat# 352350
Corning 125mL Erlenmeyer Flask with vent cap	Corning	Cat# 431143
Costar® Ulta-low attachemnt 96 well plate	Corning	Cat# 7007
Costar® Ulta-low attachemnt 6 well plate	Corning	Cat# 3471
Costar® Ulta-low attachemnt 24 well plate	Corning	Cat# 3473
Costar® 24 well clear TC-treated plates	Corning	Cat# 3524
Corning 75cm2 U-shaped Canted neck flask with vent	Corning	Cat# 430641U
Corning 96-well clear flat bottom polystyrene plates	Corning	Cat# 3598
24-well plate with Gold Electrodes	MultiChannel Systems	Cat# 24W700/100F-28
Cover glass #1.5, 22x60mm	Ted Pella	Cat# 260154-60
Superfrost Plus Microscope Slides	Thermo Fisher	Cat# 22-037-246
Equipment		
BioTek Plate Reader	Agilent	Model# Synergy 4
FACSymphony Cell Analyzer	BD Biosciences	Model# A5
Mastercycler-PCR thermal cycler	Eppendorf	Model# nexus GX2
Digital Orbital Shaker	Health Care Logistics	Item# 20190
Luminex 200 Instrument System	Invitrogen	Item# APX100310
Aperio Versa 8 slide scanner	Leica Biosystems	Model# Versa 8
Leica Cryostat	Leica Biosystems	Model# CM3050S
Multiwell-MEA-System	MultiChannel Systems	https://www.multichannelsystems.com/
iBright Imaging System	Thermo Fisher	Model# 1500
QuantStudio Real-Time PCR System	Thermo Fisher	Model# 7 PRO
NanoDrop One	Thermo Fisher	Model# ND-ONE-W
