# Supplementary material for: Proteasome mutations associated with CANDLE syndrome cause altered neuronal development by dysregulating polyamine synthesis
Source: J Clin Invest. 2026 Aug 4;136(19):e199140. doi: 10.1172/JCI199140 (PMC13626833; doi:10.1172/JCI199140)
Supplement: Supplementary file 2 [file jci-136-199140-s270.pdf]

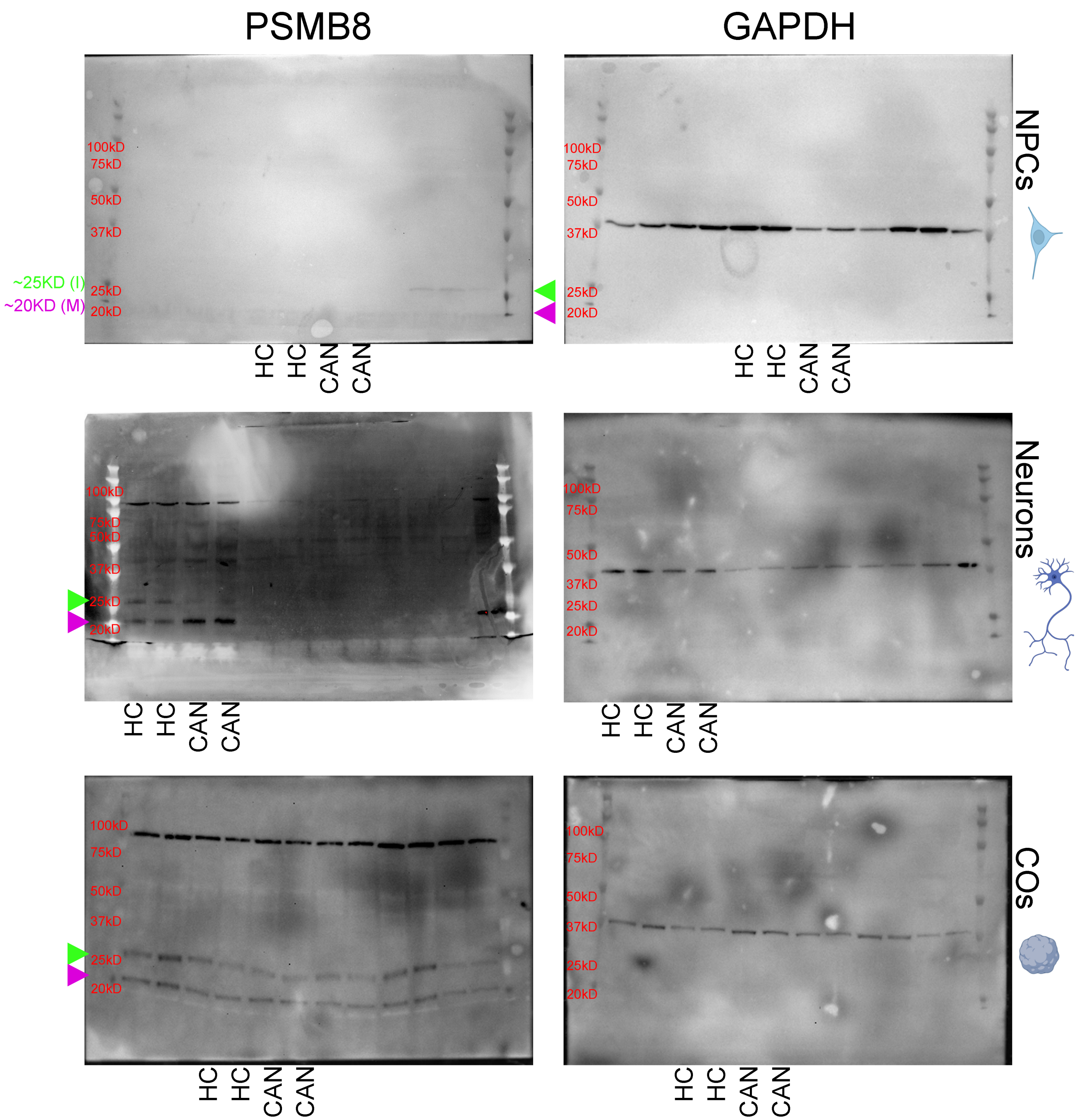

Whole western blots images of PSMB8 expression in iPSC-derived NPCs, neurons and COs. Western Blot analysis for PSMB8 expression by NPCs (top, left column), neurons (middle, left column) and COs (bottom, left column) generated from the iPSC-derived donor samples. The immature form of PSMB8 (upper green arrow) is ~26kD in molecular weight and the mature form is ~21kD (lower magenta arrow). The same blots were reprobed for GAPDH expression at ~39kD (blots in right column). Lanes for the representative cropped bands shown in Figure 6F are indicated by the labels at the bottom of each blot.
